# Tenofovir versus entecavir on the prognosis of hepatitis B virus-related hepatocellular carcinoma: a reconstructed individual patient data meta-analysis

**DOI:** 10.3389/fphar.2024.1393861

**Published:** 2024-08-22

**Authors:** Jian-Xin Peng, Ling-Zhi Wang, Qiu-Ting Wang, Hui-Long Li, Li-Jun Lin, Jun-Ming He

**Affiliations:** ^1^ Department of Hepatobiliary Surgery, Guangdong Provincial Hospital of Chinese Medicine, Guangzhou, China; ^2^ Department of Anesthesia, The Second Affiliated Hospital of Guangzhou Medical University, Guangzhou, China

**Keywords:** hepatocellular carcinoma, hepatitis B, tenofovir, entecavir, overall survival rates, recurrence-free survival rate, meta-analysis

## Abstract

**Background:** Hepatitis B, often leading to Hepatocellular carcinoma (HCC), poses a major global health challenge. While Tenofovir (TDF) and Entecavir (ETV) are potent treatments, their comparative effectiveness in improving recurrence-free survival (RFS) and overall survival (OS) rates in HBV-related HCC is not well-established.

**Methods:** We conducted an individual patient data meta-analysis using survival data from randomized trials and high-quality propensity score-matched studies to compare the impact of Tenofovir (TDF) and Entecavir (ETV) on RFS and OS in HBV-related HCC patients. Data from six databases and gray literature up to 30 August 2023, were analyzed, utilizing Kaplan-Meier curves, stratified Cox models, and shared frailty models for survival rate assessment and to address between-study heterogeneity. The study employed restricted mean survival time analysis to evaluate differences in RFS and OS between TDF-treated and ETV-treated patients. Additionally, landmark analyses compared early (<2 years) and late (≥2 years) tumor recurrence in these cohorts.

**Results:** This study incorporated seven research articles, covering 4,602 patients with HBV-related HCC (2,082 on TDF and 2,520 on ETV). Within the overall cohort, TDF recipients demonstrated significantly higher RFS (*p* = 0.042) and OS (*p* < 0.001) than those on ETV. The stratified Cox model revealed significantly improved OS for the TDF group compared to the ETV group (hazard ratio, 0.756; 95% confidence interval, 0.639–0.896; *p* = 0.001), a result corroborated by the shared frailty model. Over a follow-up period of 1–8 years, no significant difference was noted in the mean time to death between the TDF and ETV groups. The rates of early recurrence did not significantly differ between the groups (*p* = 0.735). However, TDF treatment was significantly associated with a reduced risk of late recurrence compared to ETV (*p* < 0.001). In the HCC resection subgroup, the disparities in OS, early, and late recurrence rates between the two treatments paralleled those seen in the overall cohort.

**Conclusion:** Compared to ETV, TDF may enhance OS and reduce late tumor recurrence risk in HBV-related HCC patients receiving curative treatment. However, there was no statistically significant distinction in the timing of tumor recurrence and mortality between patients administered TDF and those prescribed ETV.

**Systematic Review Registration:** http://www.crd.york.ac.uk/prospero/.

## Introduction

Hepatitis B is a global health concern, affecting approximately 296 million people worldwide with chronic hepatitis B infection (Hsu et al., 2023). Hepatocellular carcinoma (HCC) is one of the primary and most lethal outcomes of chronic hepatitis B (CHB). It is the third leading cause of cancer-related mortality globally and it is estimated that around 830,000 deaths occur annually worldwide due to HCC (Vogel et al., 2022). Despite the introduction of numerous treatment modalities over the past few decades, including hepatic resection, liver transplantation, ablative therapies, transarterial chemoembolization, radiotherapy, and systemic antineoplastic treatments (Zhou et al., 2023), it is unfortunate that a high level of hepatitis B virus (HBV) DNA remains an independent risk factor for the recurrence of HCC, even in cases undergoing curative liver resection, consequently leading to diminished postoperative survival rates (Wang et al., 2020). Previous research has demonstrated that nucleos(t)ide analogues (NAs) treatment not only significantly reduces the incidence of HBV-related HCC (Wu et al., 2014), but also markedly prolongs the overall survival of patients with HBV-related HCC and reduces tumor recurrence by lowering viral load (Huang et al., 2015; Lee et al., 2016). While NAs therapy contributes to improved prognoses in HBV-related HCC, it fails to cure HBV or completely prevent HCC recurrence. This is attributed to the inability of NAs to eliminate covalently closed circular DNA (cccDNA) within HBV-infected hepatocytes, which can persist and potentially reactivate within the liver cells (Yang and Kao, 2014). Until the advent of novel antiviral agents targeting cccDNA or hepatocytes harboring cccDNA, optimizing NAs therapy remains a primary focus.

Tenofovir (TDF) and Entecavir (ETV), as potent NAs with high resistance barriers, are recommended in major clinical guidelines as primary treatments for chronic HBV infection (Sarin et al., 2016; Lampertico et al., 2017; Terrault et al., 2018; You et al., 2023a). Although both TDF and ETV have similar antiviral efficacy, their relative impacts on the prognosis of HBV-related HCC patients are debated. Recent meta-analysis suggests that compared with ETV, TDF has the advantage of improving recurrence-free survival (RFS) and overall survival (OS) of patients with HBV-related HCC who underwent resection (Kong et al., 2023; Liu et al., 2023). However, this analysis is limited by its reliance on aggregated study-level data, which overlooks individual patient differences, and by the lack of individual patient time-to-event data, making it impossible to accurately calculate survival rates and risks at each time point, thus affecting the precision of RFS and OS estimates. To address these limitations, this study conducts an individual patient data meta-analysis (IPDMA) using randomized controlled trial or high-quality propensity score-matched cohort study data. This approach provides more accurate RFS and OS estimates and resolves ongoing debates. IPDMA is considered the gold standard for pooled analysis of time-to-event data, as it comprehensively accounts for censoring and effectively addresses both between-study and within-study heterogeneity (De Jong et al., 2020). Importantly, it also allows for testing violations of the proportional hazards assumption, an aspect not feasible in traditional meta-analyses (Rahman et al., 2019).

## Methods

### Search strategy and selection criteria

We conducted a literature synthesis in accordance with the Preferred Reporting Items for Systematic Reviews and Meta-Analyses (PRISMA) guidelines for individual participant data systematic reviews (Stewart et al., 2015; Page et al., 2021). Six databases (Web of Science, PubMed, Embase, Scopus, Cochrane CENTRAL, LILACS) and gray literature (OpenGrey, ProQuest Dissertations and Theses Global) were searched without language restriction from inception to 30 August 2023. Keywords and Medical Subject Headings (MeSH) terms pertaining to HBV-related HCC, TDF, and ETV were integrated into the search strategy. Details of the full search strategy are available in Supplementary Methods. Two reviewers independently screened titles and abstracts and subsequently performed full-text reviews. Any disagreements were resolved through discussion, involving a senior reviewer when necessary. The inclusion criteria for this study were patients diagnosed with HBV-related HCC, studies comparing the effects of TDF *versus* ETV, and reporting outcomes as RFS and/or OS. The study types included randomized controlled trials or high-quality PSM studies. Sufficient data were required, including Kaplan-Meier survival curves, time-to-event data, and detailed patient characteristics (Tan et al., 2022)**.** Studies employing other confounder control methods, including covariance adjustment, stratification, and inverse probability of treatment weighting, were excluded. Although these techniques are typically effective in reducing bias, their balancing effect is lost in meta-analyses using reconstructed individual patient data, since meta-analysts are unaware of the patient-level covariates or propensity scores used for bias control (Syn et al., 2021). For studies with overlapping patient populations in multiple papers, we selected the article that provided the most data with either the largest patient sample, the most subgroup data, and/or the most updated data.

### Data extraction and study quality assessment

Two reviewers independently extracted study characteristics, encompassing patient demographics, tumor profiles, and biochemical parameters, in addition to covariates employed for propensity score matching. Disagreements were resolved through discussion, in cases where consensus was not reached, a senior reviewer served as an arbitrator. Individual patient data were reconstructed from published survival curves utilizing the methodology proposed by Guyot and colleagues (Guyot et al., 2012). Additionally, two reviewers independently evaluated the quality of included studies employing the Newcastle-Ottawa Scale for cohort studies (Wells et al., 2023), with any disagreements resolved through consensus or consultation with a senior reviewer.

### Statistical analysis

All analyses were conducted using R statistical software (version 4.2.3, R Foundation for Statistical Computing, 2023). For the baseline characteristics included in the study (if data is available), dichotomous variables were pooled using means and 95% confidence intervals. When median data were reported, they were converted to means and standard deviations using established methodologies prior to being pooled (Wan et al., 2014; Luo et al., 2018). For continuous variables, the data were pooled using constituent ratios and 95% confidence intervals. The heterogeneity of the included studies was assessed using Cochran’s Q statistic and the *I* ^2^ metric. When *I* ^2^ was less than 50%, a fixed-effect model was employed; otherwise, a random-effect model was utilized for pooling the results. Kaplan-Meier curves were used to plot the RFS rates and OS rates of HBV-related HCC, with intergroup differences assessed using the log-rank test. Sensitivity analysis was also conducted to evaluate the stability of the results using a “leave-one-out” analysis. We employed both stratified Cox models and shared frailty models to address between-study heterogeneity. Both models need to satisfy the proportional hazards assumption and cannot directly handle time-dependent covariates. We used the Grambsch-Therneau test to assess the proportionality assumption and visually assessed the non-zero slope using Schoenfeld residuals. The restricted mean survival time (RMST) analysis evaluated differences in RFS and OS between patients who received TDF and those who received ETV over time. Landmark analyses were conducted based on a prespecified landmark point at 2 years, comparing early (<2 years) and late (≥2 years) tumor recurrence between patients treated with TDF and ETV, respectively (Yoo et al., 2022). Risk of publication bias was evaluated with a Funnel plot, Begg’s rank correlation test, and Egger’s regression test.

## Results

### Summary of included articles

**FIGURE 1:**
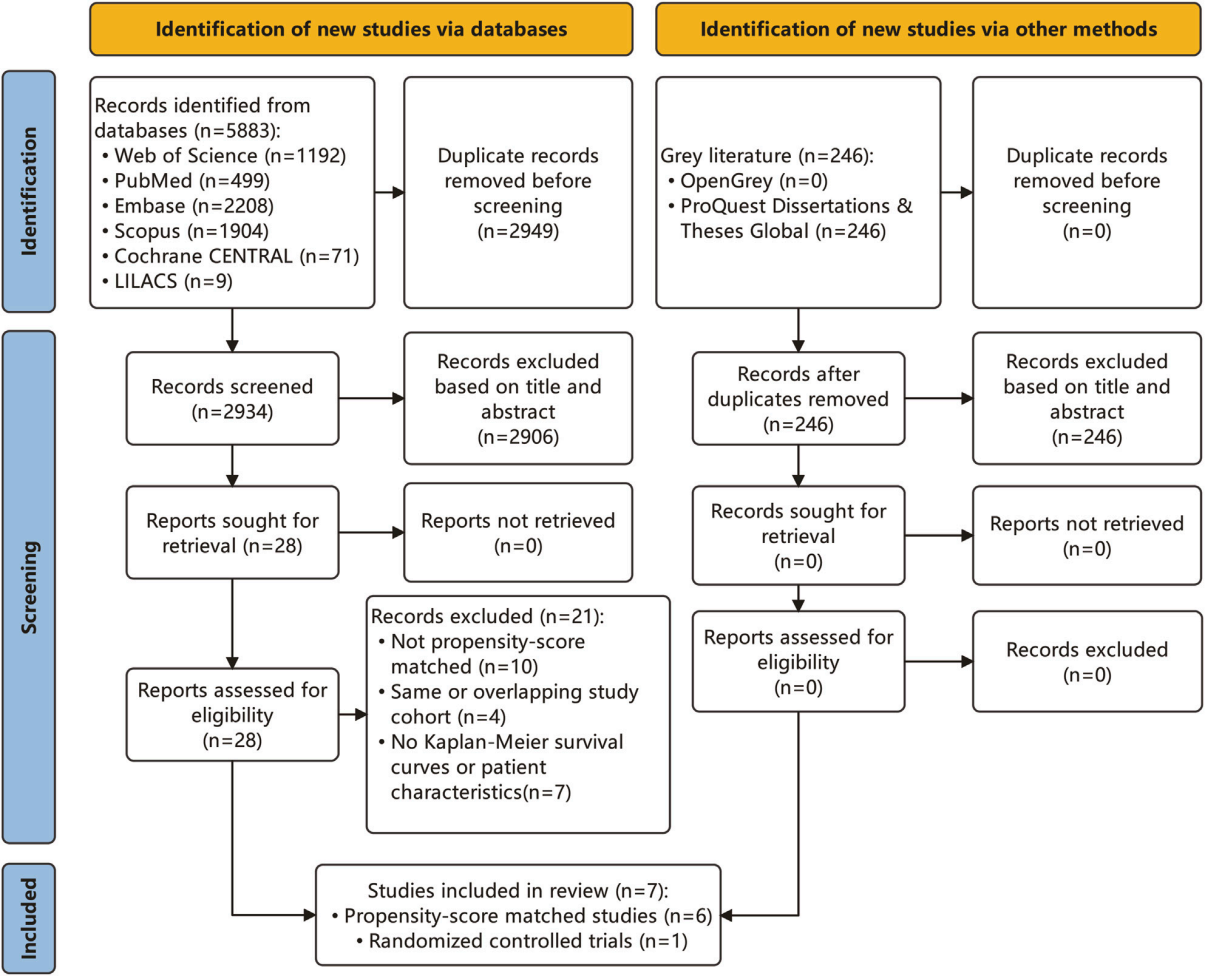
PRISMA flowchart of the study selection.

A comprehensive search across six databases identified 5,883 articles (Figure 1). After duplicate removal, 2,934 articles remained for consideration. Subsequent title and abstract review led to the exclusion of 2,906 articles, leaving 28 for full-text evaluation. Additionally, a gray literature search revealed 246 articles, with none meeting the inclusion criteria. Eventually, seven articles (Qi et al., 2021; Hu et al., 2022; Tsai et al., 2022; Wang et al., 2022; Yun et al., 2022; Linye et al., 2023; Yang et al., 2023) fulfilled the study’s inclusion criteria and were incorporated into the analysis, as detailed in Supplementary Table S1. These studies, published between 2021 and 2023, included five conducted in mainland China (Qi et al., 2021; Hu et al., 2022; Wang et al., 2022; Linye et al., 2023; Yang et al., 2023), one in Taiwan (Tsai et al., 2022), and one in South Korea (Yun et al., 2022). The study designs comprised six retrospective studies and one randomized controlled trial. Four studies were single-center, and the other three were multi-center, with two deriving from administrative databases and one from a clinical cohort. The patient interventions in these studies varied: five studies conducted HCC resection surgery (Qi et al., 2021; Tsai et al., 2022; Wang et al., 2022; Yun et al., 2022; Linye et al., 2023), and two implemented treatments other than HCC resection (Hu et al., 2022; Yang et al., 2023), including liver transplantation and radiofrequency ablation (RFA). The original and reconstructed survival curves from these studies are depicted in Supplementary Figure S1. The quality of the included studies was high, with each scoring 8 or higher on the Newcastle Ottawa Scale, as shown in Supplementary Table S2. Visual inspection of funnel plots, along with Begg’s rank correlation test and Egger’s regression test, indicated no evidence of publication bias (Supplementary Figure S2).

### Baseline characteristics

Table 1 outlines the baseline characteristics of HBV-related HCC patients who received TDF and those who underwent ETV treatment following propensity score matching. The characteristics included a mean age of 52.42 (95% confidence interval [CI], 50.87–53.97) years and a sex distribution of 85.96% (95% CI, 83.43%–88.50%) male and 14.04% (95% CI, 11.50%–16.57%) female. No significant differences in these characteristics were noted between the TDF and ETV groups. The median follow-up period was 39.92 (IQR, 27.2–55.84) months for the TDF group and 45.77 (IQR, 28.32–63.94) months for the ETV group.

**TABLE 1:** Summary of baseline characteristics comparing patients receiving TDF vs ETV.

Characteristics	Number of studies	TDF cohort		ETV cohort		*p*-value
		Total number of patients	Value (95% CI)	Total number of patients	Value (95% CI)	
Basic characteristics						
Age, year, mean	7	2082	52.38 (50.15–54.60)	2,520	52.44 (50.11–54.76)	0.385
Sex, %						0.087
Male	7	2082	86.36 (82.72–90.00)	2,520	85.68 (81.94; 89.42)	
Female	7	2082	13.64 (10.00–17.28)	2,520	14.32 (10.58–18.06)	
Hypertension, %	5	1971	19.74 (10.25–29.22)	2,306	21.20 (13.83–28.57)	0.978
Diabetes, %	6	2044	13.89 (8.49–19.28)	2,452	15.87 (11.57–20.17)	0.396
Liver cirrhosis, %	6	2044	69.59 (59.09–80.08)	2,452	68.58 (57.29–79.86)	0.814
Tumor characteristics						
Tumor size, cm, mean	6	671	4.21 (2.31–6.12)	1,109	4.20 (2.35–6.04)	0.917
Singe tumor, %	5	633	87.45 (79.46–95.45)	1,041	87.63 (84.29–90.98)	0.848
Microvascular invasion, %	5	594	28.37 (21.92–34.82)	979	31.39 (22.70–40.08)	0.483
Satellite nodule, %	4	329	6.37 (3.75–8.99)	576	7.80 (3.46–12.15)	0.930
BCLC stage 0, %	5	633	16.23 (1.21–31.24)	1,041	12.31 (2.02–22.61)	0.114
High differentiation	5	594	10.44 (3.45–31.62)	979	7.81 (1.80–33.97)	0.998
Virologic characteristics						
HBV-DNA ≥2000 IU/mL, %	3	412	40.33 (25.56–55.09)	623	40.24 (23.29–57.18)	0.814
HBeAg positive, %	4	556	23.78 (18.87–28.70)	911	23.73 (17.85–29.62)	0.937
Laboratory Test						
AFP >20 ng/mL, %	4	489	52.30 (47.28–57.33)	753	51.20 (45.21–57.20)	0.885
PLT, 10^9^/L, mean	4	560	157.02 (102.11–211.93)	895	155.70S (96.62–214.79)	0.683
ALT, U/L, mean	5	633	43.80 (34.54–53.06)	1,041	43.29 (34.12–52.46)	0.725
AST, U/L, mean	5	633	41.86 (31.86–51.86)	1,041	41.27 (31.26–51.28)	0.548
ALB, g/L, mean	6	671	41.21 (39.03–43.40)	1,109	40.90 (38.80–42.99)	0.271
TBIL, μmol/L, mean	6	671	14.52 (13.92–15.12)	1,109	16.37 (13.19–19.55)	0.766
Cr, μmol/L, mean	3	376	75.91 (70.29–81.52)	617	75.01 (72.08–77.95)	0.800
PT, s, mean	4	524	13.22 (11.37–15.07)	889	13.20 (11.57–14.83)	0.896

TDF, tenofovir; ETV, entecavir; CI, confidence interval; BCLC, barcelona clinic liver cancer; HBV, hepatitis B virus; HBeAg, hepatitis B e antigen; AFP, alpha-fetoprotein; PLT, platelet count; ALT, alanine aminotransferase; AST, aspartate aminotransferase; ALB, albumin; TBIL, total bilirubin; Cr, creatinine; PT, prothrombin time.

### Analysis of the overall cohort

#### RFS and OS in overall cohort

**FIGURE 2:**
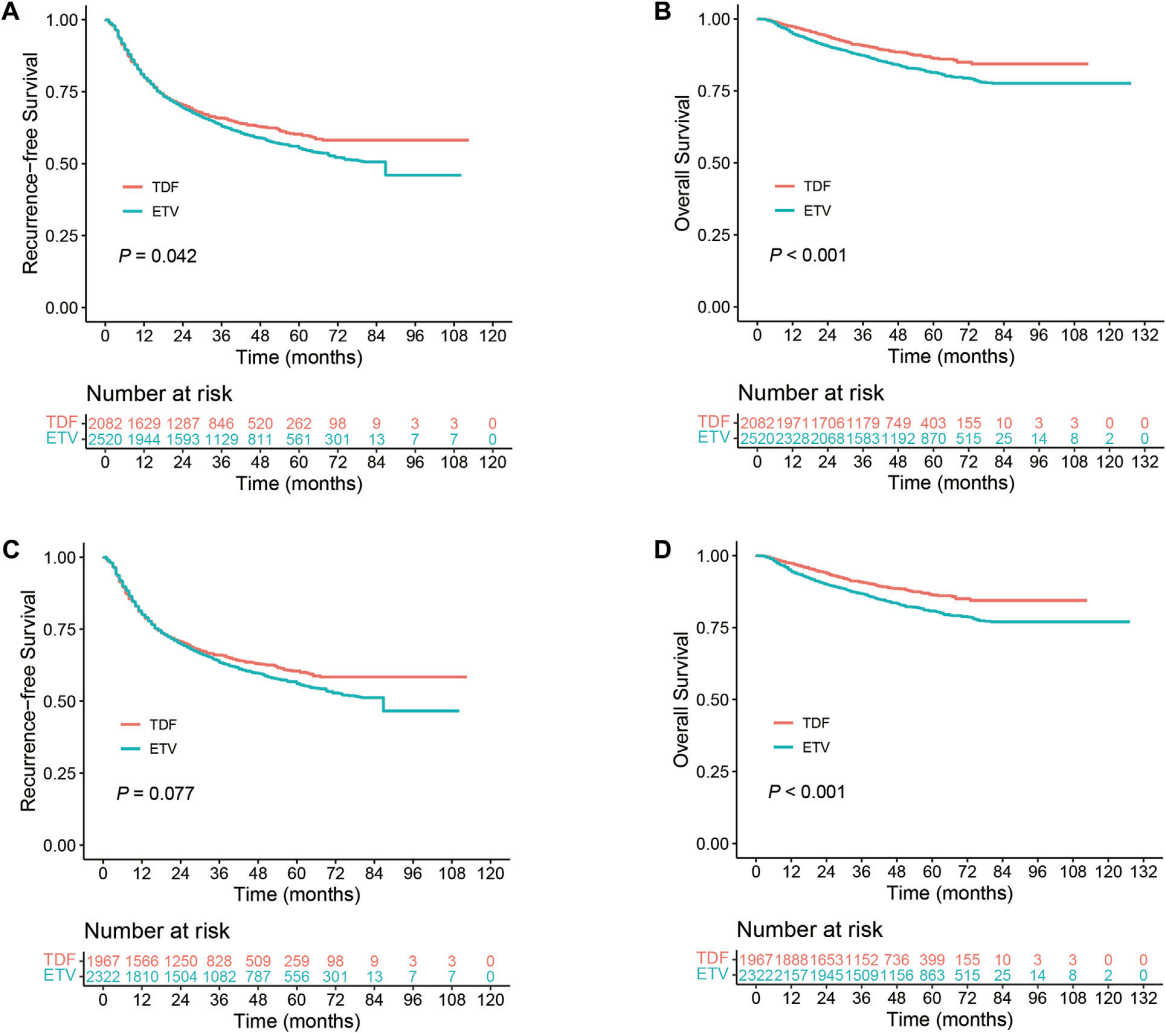
Comparison of TDF and ETV in HBV-related HCC patients. **(A)** RFS in the overall cohort; **(B)** OS in the overall cohort; **(C)** RFS in the Resection Subgroup; **(D)** OS in the Resection Subgroup.

In the overall cohort, our analysis encompassed 4,602 patients with HBV-related HCC from seven studies, comprising 2,082 patients treated with TDF and 2,520 with ETV. The 1-, 3-, and 5-year RFS rates for the TDF group were 80.5% (95% CI, 78.8%–82.3%), 65.8% (95% CI, 63.7%–68.0%), and 60.2% (95% CI, 57.8%–62.8%), respectively. Conversely, the ETV group reported RFS rates of 80.2% (95% CI, 78.6%–81.8%), 63.5% (95% CI, 61.6%–65.5%), and 55.6% (95% CI, 53.5%–57.9%). Patients receiving TDF antiviral therapy demonstrated a significantly higher RFS compared to those on ETV therapy (*p* = 0.042, Figure 2A). The OS rates at these intervals for the TDF group were 97.4% (95% CI, 96.7%–98.1%), 90.8% (95% CI, 89.5%–92.2%), and 86.5% (95% CI, 84.6%–88.4%), while the ETV group had OS rates of 95.2% (95% CI, 94.4%–96.1%), 87.4% (95% CI, 86.0%–88.7%), and 81.4% (95% CI, 79.7%–83.2%). The TDF group exhibited a markedly improved OS compared to the ETV group (*p* < 0.001, Figure 2B). A sensitivity analysis verified the robustness of the RFS and OS outcomes (Supplementary Figure S3A,B). In the stratified Cox model, which accounts for inter-study heterogeneity, the OS was significantly greater in the TDF group compared to the ETV group (hazard ratio [HR], 0.756; 95% CI, 0.639–0.896; *p* = 0.001). Subsequent analysis using the shared frailty model produced similar findings (Table 2). Regarding RFS, the proportionality assumption was not satisfied (*p* = 0.001); consequently, analyses using the stratified Cox model and the shared frailty model were not conducted. The RMST analysis assessed differences in RFS and OS among patients receiving TDF *versus* those receiving ETV in the overall cohort over time. This analysis found no significant differences in the mean time to recurrence between the TDF and ETV groups during a follow-up period of 1–8 years (RMST difference at 1 year, −0.001 years [95% CI, −0.002–0.001], *p* = 0.404; RMST difference at 8 years, −0.025 years [95% CI, −0.103–0.053], *p* = 0.528) (Supplementary Table S3). Similarly, no significant differences were observed in the mean time to death between the TDF and ETV groups over the same period (RMST difference at 1 year, 0.000 years [95% CI, −0.001–0.000], *p* = 0.317; RMST difference at 8 years, 0.022 years [95% CI, −0.004–0.048], *p* = 0.092) (Supplementary Table S4).

**TABLE 2:** Summary of the analysis on early and late recurrence and OS in patients treated with TDF and ETV.

Variable	Early recurrence		Late recurrence		OS	
	HR (95% CI)	*p*-value	HR (95% CI)	*p*-value	HR (95% CI)	*p*-value
Overall cohort						
Stratified Cox regression	1.045 (0.937–1.165)	0.434	0.681 (0.550–0.843)	<0.001	0.756 (0.639–0.896)	0.001
Shared frailty model	1.040 (0.933–1.160)	0.480	0.690 (0.558–0.853)	<0.001	0.753 (0.636–0.891)	0.001
Resection cohort						
Stratified Cox regression	1.050 (0.938–1.176)	0.393	0.691 (0.556–0.859)	0.001	0.736 (0.620–0.874)	<0.001
Shared frailty model	1.047 (0.935–1.172)	0.420	0.701 (0.565–0.870)	0.001	0.731 (0.616–0.868)	<0.001

OS, Overall survival; TDF, tenofovir; ETV, entecavir; CI, confidence interval.

### Early and late recurrence in overall cohort

HCC recurrence is commonly classified as either early or late, typically defined by a 2-year cutoff following surgery (Imamura et al., 2003; Wu et al., 2009). We conducted a landmark analysis to evaluate recurrence in HBV-related HCC patients from the overall cohort who received TDF or ETV. Supplementary Figure S4A illustrates that early recurrence rates did not significantly differ between the groups (*p* = 0.735). However, TDF treatment, in comparison to ETV, was significantly associated with a reduced risk of late recurrence (*p* < 0.001). The stratified Cox model revealed no significant difference in early tumor recurrence between the TDF and ETV groups (HR, 1.045; 95% CI, 0.937–1.165; *p* = 0.434). However, TDF was associated with a significantly lower risk of late tumor recurrence compared to ETV (HR, 0.681; 95% CI, 0.550–0.843; *p* < 0.001). Analysis employing the shared frailty model yielded consistent results (Table 2).

### Analysis of the resection subgroup

#### RFS and OS in resection subgroup

In the resection subgroup, our study included 4,289 patients with HBV-related HCC, with 1,967 receiving TDF and 2,322 undergoing ETV therapy. RFS rates in the TDF group at 1-, 3-, and 5-year were 80.7% (95% CI, 79.0%–82.5%), 66.0% (95% CI, 63.9%–68.2%), and 60.5% (95% CI, 58.0%–63.1%), respectively. In the ETV group, these rates were 80.4% (95% CI, 78.8%–82.1%), 64.0% (95% CI, 62.0%–66.1%), and 56.3% (95% CI, 54.1%–58.7%). The differences in recurrence-free survival between the therapies were not statistically significant. However, TDF therapy demonstrated a potential improvement in RFS (*p* = 0.077, Figure 2C). In terms of OS, the 1-, 3-, and 5-year rates for the TDF group were 97.4% (95% CI, 96.7%–98.1%), 90.8% (95% CI, 89.5%–92.2%), and 86.5% (95% CI, 84.6%–88.5%). For the ETV group, the rates were 94.9% (95% CI, 93.9%–95.8%), 86.9% (95% CI, 85.4%–88.3%), and 80.8% (95% CI, 79.0%–82.6%). OS was significantly higher in patients treated with TDF compared to ETV (*p* < 0.001, Figure 2D). A sensitivity analysis confirmed the robustness of the RFS and OS findings in the resection subgroup (Supplementary Figure S3C,D). In the stratified Cox model, a significant increase in OS was observed in the TDF group compared to the ETV group (HR, 0.736; 95% CI, 0.620–0.874; *p* < 0.001). Analysis using the shared frailty model revealed similar results (Table 2). Concerning RFS, the proportionality assumption was not met; therefore, analyses employing the stratified Cox model and the shared frailty model were not performed. The RMST analysis showed no significant differences in the mean time to recurrence between the TDF and ETV groups, even after 8 years of follow-up (RMST difference at 8 years, −0.030 [95% CI, −0.11–0.049] years; *p* = 0.454). The mean time to death also exhibited a similar pattern (Supplementary Table S4).

#### Early and late recurrence in resection subgroup

As shown in Supplementary Figure S4B, the landmark analysis of recurrence in HBV-related HCC patients within the resection subgroup, treated with either TDF or ETV, revealed no significant difference in early recurrence rates between the groups (*p* = 0.866). However, TDF treatment, as opposed to ETV, significantly correlated with a lower risk of late recurrence (*p* < 0.001). The stratified Cox regression analysis showed no significant disparity in early tumor recurrence rates between the TDF and ETV groups (HR, 1.050; 95% CI, 0.938–1.176; *p* = 0.393). In contrast, TDF treatment was significantly associated with a reduced risk of late tumor recurrence compared to ETV (HR, 0.691; 95% CI, 0.556–0.859; *p* = 0.001). The application of the shared frailty model corroborated these findings, as detailed in Table 2.

## Discussion

In this study, we conducted a meta-analysis using reconstructed IPD from 4,602 patients with HBV-related HCC. Among these patients, 2,082 received TDF treatment, while 2,520 received ETV treatment. The aim was to investigate the impact of TDF compared to ETV on the RFS and OS of individuals with HBV-related HCC. Given the absence of multicenter, large-sample randomized controlled clinical trials, this analysis represents the most robust evidence available to date for assessing the effects of TDF vs ETV on the prognosis of patients with HBV-related HCC. In the overall cohort, we observed that patients treated with TDF had a lower risk of tumor recurrence and higher overall survival rates compared to those treated with ETV. These findings align with previous meta-analysis results (Liu et al., 2023). A network meta-analysis, involving 13,517 participants from 16 studies (including 11 randomized controlled trials and five propensity-matched cohort studies), compared the efficacy of TDF and ETV in the treatment of chronic hepatitis B patients over a 48-week period. The results demonstrated that at the 48-week mark, patients receiving TDF treatment exhibited a higher virological response rate than those receiving ETV treatment, with a more pronounced difference noted in patients positive for hepatitis B e antigen (Con et al., 2021). Moreover, TDF-treated patients exhibited elevated levels of serum interferon (IFN)-λ3 (Murata et al., 2018; Umemura et al., 2022), a factor known to have the potential to directly or indirectly inhibit tumor growth (Stiff and Carson, 2015). Additionally, an *in vitro* study suggested that TDF could restore the function of T cells and natural killer cells by downregulating interleukin (IL)-10 and upregulating IL-12, thereby playing crucial roles in antiviral and antitumor immunity (MURATA et al., 2020). Research also shows that TDF can inhibit the translocation of Akt to the plasma membrane, thereby blocking the mammalian target of rapamycin pathway, which is commonly activated in most cancer cells (Murata and Mizokami, 2023). These findings offer plausible explanations for the superior prognosis associated with TDF compared to ETV in the treatment of patients with HBV-related HCC. Nevertheless, further research is warranted to elucidate these differences fully. Due to the violation of the proportional hazards assumption (*p* = 0.001), we refrained from employing stratified Cox models and shared frailty models to analyze the occurrence of HCC recurrence in the overall cohort. However, the RMST analysis revealed that by the 8 years of treatment, no statistically significant differences were observed in the times to tumor recurrence and death between patients treated with TDF and those treated with ETV, with reductions of 9 days and extensions of 8 days, respectively. The divergence between the RMST results and those of the log-rank test can be attributed to the distinct statistical properties and sensitivities of these methodologies. The log-rank test, which is sensitive to differences at any point during the follow-up period, indicated a benefit with TDF treatment. In contrast, the RMST, which calculates a summary measure of survival within a predefined period and is robust against violations of the proportional hazards assumption, suggested no significant long-term differences between the treatments over an 8-year span. This suggests that although patients receiving TDF demonstrate a decreased incidence of tumor recurrence and enhanced OS compared to those receiving ETV, the reduction in tumor recurrence time and the extension in survival time are not significant. This is consistent with the current Guidelines for the Prevention and Treatment of Chronic Hepatitis B (2022 edition), which do not explicitly prefer TDF over ETV (You et al., 2023b). Consequently, when deciding between TDF and ETV, factors such as drug suitability, comorbidities, and cost-effectiveness should take precedence. For instance, research has indicated that TDF may be a more appropriate choice for HBeAg-positive patients, especially those with high viral loads, as it demonstrates more robust suppression of HBV DNA levels (Gao et al., 2014). Additionally, it is widely acknowledged that TDF may induce nephrotoxic and osteotoxic side effects (Lim et al., 2019; Jung et al., 2022). Therefore, ETV may represent a preferable option for patients with renal impairment or osteoporosis, given that TDF could exacerbate renal or skeletal issues.

In our analysis of HCC recurrence, we categorized cases into early and late stages, utilizing a 2-year landmark point. The Grambsch-Therneau test yielded a *p*-value of 0.041 for the stratified Cox model applied to the early recurrence cohort. Although this *p*-value falls below the commonly accepted threshold of 0.05, it signifies a noteworthy enhancement when compared to the Grambsch-Therneau test’s *p*-value for the Overall Cohort. Consequently, the decision was made to utilize the stratified Cox model for our analysis. We performed log-rank tests, stratified Cox models, and shared frailty models for both early and late recurrence cohorts, consistently revealing that TDF mitigated the risk of late recurrence rather than early recurrence. This suggests that the beneficial effects of TDF on HCC may require some time to become evident. Conventionally, early recurrence is often associated with the invasiveness of the primary tumor and the presence of microvascular invasion (Poon et al., 2000; Imamura et al., 2003). The potential of TDF and ETV to reduce the risk of early recurrence in HBV-related HCC by modifying the tumor microenvironment remains uncertain and necessitates further investigation. In contrast, late recurrence is typically attributed to multicentric tumor growth or the development of *de novo* cancer (Xu et al., 2019), influenced by ongoing hepatitis B virus infection and/or cirrhosis. While NAs effectively suppress HBV-DNA replication, they fall short of completely clearing HBsAg. Research indicates that preoperative HBsAg levels exceeding 1000 IU/mL independently elevate the risk of HCC recurrence in patients with low HBV DNA levels (Huang et al., 2014). Moreover, cirrhosis represents a significant complication of HBV infection and a pivotal factor in HCC development (Rizzo et al., 2022). TDF diminishes the risk of late recurrence, potentially attributed to its superior viral suppression and liver function preservation compared to ETV (Park et al., 2017; Zheng et al., 2023). TDF positively influences liver health by maintaining a sustained and stable antiviral effect, enhancing liver function, mitigating inflammatory responses, and averting fibrosis progression (Marcellin et al., 2013; Park et al., 2017; Zheng et al., 2023). Inflammation seems to play a substantial role in both the onset and advancement of HCC (Yu et al., 2018). Recent investigations have suggested that aspirin use for ≥90 days significantly lowers HCC incidence in CHB patients (Jang et al., 2022). The concurrent utilization of aspirin and TDF may represent a promising approach, yet further research is warranted to explore this possibility in future studies.

Within the HCC resection subgroup, patients who received TDF treatment exhibited a trend toward improved RFS compared to those treated with ETV, despite the absence of a statistically significant difference in tumor recurrence risk. This hints at a potential disparity in HCC recurrence risk associated with these two treatment modalities. The discrepancies in OS, early, and late recurrence rates between the two groups closely mirrored those observed in the overall cohort. Given that all seven studies included in this analysis exclusively involved curative interventions, comprising five instances of surgical resection, one liver transplantation case, and one curative RFA procedure, the study’s conclusions are specifically relevant to patients undergoing curative treatments. Curative interventions are typically most suitable for HCC patients with smaller and limited tumors, as well as those in good liver and overall health (Cucchetti et al., 2023). Among these cases, those undergoing HCC resection surgery constituted the majority, accounting for 93.2% (4,289/4,602) of the entire cohort. Consequently, the differences between the two drugs within the overall cohort primarily reflect variations following HCC resection surgery. As only two articles discussed treatments other than liver resection surgery, data from these studies were not incorporated into the pooled analysis.

Our study addresses previous limitations in research concerning the prognosis of patients with HBV-related HCC treated with TDF vs ETV. We accomplished this by aggregating event-time data from individual patient-level datasets, exclusively incorporating randomized controlled trials and high-quality propensity score-matched studies. In contrast to prior meta-analyses on this subject, our approach takes into account issues such as patient attrition and low study quality. Notably, this study represents the inaugural meta-analysis on this subject that utilizes reconstructed individual participant data. Furthermore, we performed additional analyses using stratified Cox models and shared frailty models to elucidate heterogeneity among the studies. Additionally, we employed RMST analysis to elucidate treatment effects over time, especially considering the shorter follow-up duration in most TDF cohorts compared to ETV cohorts. In cases where the proportional hazards assumption was violated, RMST differences emerged as a popular alternative to hazard ratios, consistently yielding robust estimates (Uno et al., 2014). Nonetheless, we acknowledge several limitations in our study. First, although all included studies achieved a score of eight or higher on the Newcastle-Ottawa Scale, only one was a randomized controlled trial; the rest were retrospective cohort studies, potentially introducing selection bias (Sessler and Imrey, 2015). Despite our efforts to mitigate this by exclusively including high-quality propensity score-matched retrospective cohort studies, complete elimination of selection bias may not have been achieved (Heinze and Jüni, 2011). Additionally, all included articles focused on Asian populations, raising the possibility of regional bias. Moreover, while gender and age were balanced across all studies at baseline, equilibrium in tumor characteristics, virologic attributes, and laboratory tests was attained only in the majority, not all, of the studies. Furthermore, residual confounding factors such as the degree of liver fibrosis, surgical data, history of alcohol use, family history of HCC, virus genotype, previous use of NAs, and adherence to antiviral treatment may impact the results. However, these factors were not collected or were collected in only a few studies, making it impossible for us to further balance them. In future research, it is essential to conduct large-scale, prospective, multicenter studies involving diverse clinical populations to validate the differences in therapeutic effects of TDF vs ETV in patients with HBV-related HCC. Additionally, future studies should investigate the cost-effectiveness of TDF vs ETV to fully assess the economic impacts of these treatment methods in patients with HBV-related HCC.

## Conclusion

In summary, when compared to ETV, TDF demonstrates the potential to improve the OS of patients with HBV-related HCC undergoing curative treatment, concurrently reducing the risk of late tumor recurrence. Notably, at the 8th year of treatment, there was no statistically significant distinction in the timing of tumor recurrence and mortality between patients administered TDF and those prescribed ETV, with a reduction of 9 days and an extension of 8 days, respectively. The selection between TDF and ETV should be guided by individual patient-specific factors and convenience. However, it is imperative to underscore the necessity for additional large-scale, prospective, multicenter studies to corroborate these findings.

## Data Availability

The datasets presented in this study can be found in online repositories. The names of the repository/repositories and accession number(s) can be found in the article/Supplementary Material.
