# Supplementary material for: Tenofovir versus entecavir on the prognosis of hepatitis B virus-related hepatocellular carcinoma: a reconstructed individual patient data meta-analysis
Source: Front Pharmacol. 2024 Aug 22;15:1393861. doi: 10.3389/fphar.2024.1393861 (PMC11374766; doi:10.3389/fphar.2024.1393861)
Supplement: Supplementary file 1 [file DataSheet1.docx]

**Supplementary materials**

**Supplementary Methods.** Search strategy.

**Supplementary Table S1.** Summary of included articles.

**Supplementary Table S2.** Assessment of risk of bias in included studies (modified Ottawa-Newcastle scale).

**Supplementary Table S3.** Restricted mean survival time analysis of recurrence rate in patients treated with TDF vs. ETV.

**Supplementary Table S4.** restricted mean survival time analysis of death rate in patients treated with TDF vs. ETV.

**Supplementary Figure S1.** Reconstructed survival curves.

**Supplementary Figure S2.** Assessment of publication bias.

**Supplementary Figure S3.** Sensitivity analysis.

**Supplementary Figure S4.** Early and late recurrence in HBV-related HCC patients receiving TDF vs. ETV.

**Figure 1.** Flow Diagram

**Figure 2.** Comparison of TDF and ETV in HBV-related HCC patients.

**Figure 2A.** RFS in the overall cohort

**Figure 2B.** OS in the overall cohort

**Figure 2C.** RFS in the Resection Subgroup

**Figure 2D.** OS in the Resection Subgroup

**Supplementary Table 1.** Summary of baseline characteristics comparing patients receiving TDF vs. ETV.

**Supplementary Table 2.** Summary of the analysis on early and late recurrence and OS in patients treated with TDF and ETV.

**Supplementary Methods.** Search strategy.

**Web of Science**

#1 (TS=(hepat*)) OR TS=(liver)

#2 ((((TS=(carcinom*)) OR TS=(cancer*)) OR TS=(neoplas*)) OR TS=(malign*)) OR TS=(tumo*)

#3 TS=(HCC)

#4 (#1 AND #2) OR #3

#5 ((TS=(Tenofovir)) OR TS=(Viread)) OR TS=(TDF)

#6 ((TS=(Entecavir)) OR TS=(Baraclude)) OR TS=(ETV)

#7 #4 AND #5 AND #6

**PubMed**

#1 (Carcinoma, Hepatocellular[MeSH Terms]) OR (Liver Neoplasms[MeSH Terms])

#2 (hepat*[Title/Abstract]) OR (liver[Title/Abstract])

#3 ((((carcinom*[Title/Abstract]) OR (cancer*[Title/Abstract])) OR (neoplas*[Title/Abstract])) OR (malign*[Title/Abstract])) OR (tumo*[Title/Abstract])

#4 HCC[Title/Abstract]

#5 #1 OR (#2 AND #3) OR #4

#6 (((Tenofovir[MeSH Terms]) OR (Tenofovir[Title/Abstract])) OR (Viread[Title/Abstract])) OR (TDF[Title/Abstract])

#7 (((entecavir[Supplementary Concept]) OR (Entecavir[Title/Abstract])) OR (Baraclude[Title/Abstract])) OR (ETV[Title/Abstract])

#8 #5 AND #6 AND #7

**Embase**

#1 'liver cell carcinoma'/exp OR 'liver cell carcinoma'

#2 (('hepat*':ti,ab,kw OR 'liver':ti,ab,kw) AND ('carcinom*':ti,ab,kw OR 'cancer*':ti,ab,kw OR 'neoplas*':ti,ab,kw OR 'malign*':ti,ab,kw OR 'tumo*':ti,ab,kw)) OR 'hcc':ti,ab,kw

#3 'tenofovir'/exp OR 'tenofovir'

#4 'tenofovir':ti,ab,kw OR 'viread':ti,ab,kw OR 'tdf':ti,ab,kw

#5 'entecavir'/exp OR 'entecavir'

#6 'entecavir':ti,ab,kw OR 'baraclude':ti,ab,kw OR 'etv':ti,ab,kw

#7 (#1 OR #2) AND (#3 OR #4) AND (#5 OR #6)

**Scopus**

TITLE-ABS-KEY((("hepat*" OR "liver") AND ("carcinom*" OR "cancer*" OR "neoplas*" OR "malign*" OR "tumo*") OR "HCC") AND ("tenofovir" OR "viread" OR "tdf") AND ("entecavir" OR "baraclude" OR "etv"))

**Cochrane CENTRAL**

#1 MeSH descriptor: [Carcinoma, Hepatocellular] explode all trees

#2 MeSH descriptor: [Liver Neoplasms] explode all trees

#3 (((hepat* or liver) and (carcinom* or cancer* or neoplas* or malign* or tumo*)) or HCC):ti,ab,kw

#4 MeSH descriptor: [Tenofovir] explode all trees

#5 (Tenofovir or Viread or TDF):ti,ab,kw

#6 (Entecavir or Baraclude or ETV):ti,ab,kw

#7 (#1 or #2 or #3) and (#4 or #5) and #6

**LILACS**

tw:((((hepat* OR liver OR hepát* OR hígado OR fígado) AND (carcinom* OR cancer* OR neoplas* OR malign* OR tumo* OR cáncer* OR câncer*)) OR hcc) AND (tenofovir OR viread OR tdf) AND (entecavir OR baraclude OR etv)) AND ( db:("LILACS"))

**OpenGrey**

(((hepat* OR liver) AND (carcinom* OR cancer* OR neoplas* OR malign* OR tumo*)) OR HCC) AND (Tenofovir OR Viread OR TDF) AND (Entecavir OR Baraclude OR ETV)

**ProQuest Dissertations & Theses Global**

(((hepat* OR liver) AND (carcinom* OR cancer* OR neoplas* OR malign* OR tumo*)) OR HCC) AND (Tenofovir OR Viread OR TDF) AND (Entecavir OR Baraclude OR ETV)

**Supplementary Table S1.** Summary of included articles.

| Author, year | Country or region | Study period | Study design | Study Scale | Drug | Number of participants | Age, years (median (IQR) or mean±SD) | Male Participants n(%) | Female Participants n(%) | HCC treatment | HCC stage | Follow-up, months (median (IQR) or mean±SD) | Outcomes | Propensity score matching variables |
| --- | --- | --- | --- | --- | --- | --- | --- | --- | --- | --- | --- | --- | --- | --- |
| Yun et al, 2022 | South Korea | 2011-2017 | Retrospective study | Multicenter (National Health Insurance Service database) | Tenofovir disoproxil fumarate vs. entecavir | Before matching: 1519 vs. 2040; After matching: 1411 vs. 1411 | Before matching: 54.9±9.3 vs. 56.1±8.9; After matching: 55.0±9.3 vs. 55.4±9.2 | Before matching: 1193 (78.5) vs. 1569 (76.9); After matching: 1130 (80.1) vs. 1093 (77.5) | Before matching: 326 (21.5) vs. 471 (23.1); After matching: 281 (19.9) vs. 318 (22.5) | Resection | Naive | Naive | OS, RFS | Age, Sex, Hypertension, Diabetes, Dyslipidemia, Liver cirrhosis, Alcoholic liver disease, Metformin use, Statin use |
| Yang et al, 2023 | Chinese Mainland | 2015-2021 | Retrospective study | Single center | Tenofovir disoproxil fumarate vs. entecavir | Before matching: 44 vs. 272; After matching: 38 vs. 68 | Before matching: 50 (44-54) vs. 52 (46-59); After matching: 50 (44-55) vs. 52 (46-56) | Before matching: 41 (93.2) vs. 254 (93.4); After matching: 35 (92.1) vs. 63 (92.6) | Before matching: 3 (6.8) vs. 18 (6.6); After matching: 3 (7.9) vs. 5 (7.4) | Liver transplantation | Beyond MC or MC | Before matching: 15.5 (8.0-29.0) vs. 24.0 (13.0-39.0); After matching: 16.0 (9.2-29.0) vs. 26.0 (16.8-39.8) | OS, RFS | Age, Sex, Locoregional therapy before LT, Maximum tumor diameter, Number of lesion, Macrovascular invasion, Macrovascular tumor thrombus, Microvascular tumor invasion, Differentiation of tumor, Satellite nodule, HBV-DNA, AFP, PT, FIB, INR, TB, ALB, Cr, Valley concentration of FK506 or Ciclosporin in 1 month, Steatosis of donor liver, Cold ischemia time, MELD score |
| Wang et al, 2022 | Chinese Mainland | 2014- 2019 | Retrospective study | Single center | Tenofovir disoproxil fumarate vs. entecavir | Before matching: 349 vs. 824; After matching: 265 vs. 403 | Before matching: 49.0 (18-79) vs. 51.0 (18-80); After matching: 49.0 (18–79) vs. 49.0 (18–80) | Before matching: 302 (86.5) vs. 710 (86.2); After matching: 231 (87.2) vs. 344 (85.4) | Before matching: 47 (13.5) vs. 114 (13.8); After matching: 34 (12.8) vs. 59 (14.6) | Resection | BCLC stage 0 to B | Before matching: median 29.6 vs. 42.0; After matching: Naive | OS, RFS | Sex, Age, Diabetes, Hypertension, Tumor number, Tumor diameter, Capsule, BCLC stage, Blood loss, Transfusion, Surgical margin, Complication, Differentiation, Microvascular invasion, Cirrhosis, HBeAg, HBV-DNA, AFP, Platelet, Hemoglobin, AST, ALT, CRE, GGT, ALB, TBIL, PT |
| Tsai et al, 2022 | Chinese Taiwan | 2010-2019 | Retrospective study | Multicenter (Chang Gung Research Database) | Tenofovir disoproxil fumarate vs. entecavir | Before matching: 84 vs. 347; After matching: 73 vs. 146 | Before matching: 56.9±10.5 vs. 56.6±10.4; After matching: 56.5±10.6 vs. 56.4±10.9 | Before matching: 75 (89.3) vs. 299 (86.2); After matching: 64 (87.7) vs. 127 (87.0) | Before matching: 9 (10.7) vs. 48 (13.8); After matching: 9 (12.3) vs. 19 (13.0) | Resection | BCLC stage 0 or A | Before matching: 51.6±23.8 vs. 53.8±29.8; After matching: 52.5±24.0 vs. 52.0±28.6 | OS, RFS | Age, Sex, BMI, Diabetes, Statin use, Alcohol drinking, Smoking, Cirrhosis, BCLC stage, Tumor size, Multiple tumors, Histology grade, Microvascular invasion, Capsule invasion, Satellite nodule |
| Qi et al, 2021 | Chinese Mainland | 2014-2019 | Retrospective study | Multicenter | Tenofovir disoproxil fumarate vs. entecavir | Before matching: Naive; After matching: 144 vs. 288 | Before matching: Naive; After matching: 49.9±10.7 vs. 49.3±10.6 | After matching: 122 (84.7) vs. 247 (85.7) | Before matching: Naive; After matching: 22 (15.3) vs. 41 (14.3) | Resection | BCLC stage 0 to C | Naive | OS, RFS | Age, Sex, BCLC stage, Tumor size, Tumor number, Microvascular invasion, Diabetes, Hypertension, HBV-DNA, HBsAg, AFP, PT, RBC, Hb, WBC, PLT, AST, ALT, ALB, TBIL |
| He et al, 2023 | Chinese Mainland | 2017-2019 | Randomized controlled trial | Single center | Tenofovir disoproxil fumarate vs. entecavir | ITT population: 74 vs. 74; PP population: 72 vs. 70 | ITT population: 50.97±12.17 vs. 49.78±11.95; PP population: 50.79±12.23 vs. 49.20±11. 62 | ITT population: 63 (85.1) vs. 66 (89.2); PP population: 61 (84.7) vs. 62 (88.6) | ITT population: 11 (14.9) vs. 8(10.8); PP population: 11 (15.3) vs. 8 (11.4) | Resection | BCLC stage 0 or A | ITT population: 46.61 (18.0-62.0); PP population: 45.91 (10.0-62.0). | OS, RFS | Naive |
| Hu et al, 2022 | Chinese Mainland | 2015-2021 | Retrospective study | Single center | Tenofovir disoproxil fumarate vs. entecavir | Before matching: 102 vs. 202; After matching: 77 vs. 130 | Before matching: 53.5 (46-61.8) vs. 53 (46-63); After matching: 54 (46-62) vs. 53 (46.25-64) | Before matching: 95 (93) vs. 171 (85); After matching: 71 (92) vs. 112 (86) | Before matching: 7 (7) vs. 31 (15); After matching: 6 (8) vs. 18 (14) | Radiofrequency ablation | BCLC stage A or B | Before matching: 34.1 (19.6-47.4); After matching: Naive | OS, RFS | Age, Sex, Diabetes, Hypertension, Complications, Cirrhosis, Tumor size, Tumor number, PLT, PT, APTT, ALB, TBIL, ALT, AST, HBV-DNA, AFP, Child-Pugh class, ALBI grade |

IQR, interquartile range; SD, standard deviation; HCC, hepatocellular carcinoma; OS, overall survival; RFS, recurrence-free survival; MC, Milan criteria; LT, liver transplantation; HBV, hepatitis B virus; PT, prothrombin time; FIB, fibrinogen; INR, international normalized ratio; TB, total bilirubin; Cr, creatinine; FK506, Tacrolimus; MELD, model for end-stage liver disease; AFP, alpha-fetoprotein; AST, aspartate aminotransferase; ALT, alanine aminotransferase; CRE, creatinine; GGT, gamma-glutamyl transferase; ALB, albumin; TBIL, total bilirubin; BCLC, Barcelona clinic liver cancer; BMI, body mass index; HBsAg, hepatitis B surface antigen; RBC, red blood cell; Hb, hemoglobin; WBC, white blood cell; PLT, platelet count; ITT, intention to treat; PP, per protocol; APTT, activated partial thromboplastin time; ALBI, albumin-bilirubin.

**Supplementary Table S2.** Assessment of risk of bias in included studies (modified Ottawa-Newcastle scale).

| Author, year | Selection  (# of stars) | Comparability^a^  (# of stars) | Outcome  (# of stars) | Total  (# of stars) |
| --- | --- | --- | --- | --- |
| Yun et al, 2022 | 4 | 2 | 3 | 9 |
| Yang et al, 2023 | 3 | 2 | 3 | 8 |
| Wang et al, 2022 | 3 | 2 | 3 | 8 |
| Tsai et al, 2022 | 4 | 2 | 3 | 9 |
| Qi et al, 2021 | 4 | 2 | 3 | 9 |
| He et al, 2023 | 3 | 2 | 3 | 8 |
| Hu et al, 2022 | 3 | 2 | 3 | 8 |

^a^ One star was provided in this category if the study controlled/adjusted for any of the following: demographic characteristics(defined as age, sex), chronic disease (defined as hypertension, diabetes, liver cirrhosis), tumor characteristics (defined as tumor size, number of tumors, microvascular invasion, satellite nodule, BCLC stage, differentiation).

**Supplementary Table S3.** Restricted mean survival time analysis of recurrence rate in patients treated with TDF vs. ETV.

| Follow-up year | RMST | | RMST difference (95% CI) | *P*-value | RMST ratio (95% CI) | *P*-value |
| --- | --- | --- | --- | --- | --- | --- |
|  | TDF | ETV |  |  |  |  |
| Overall cohort |  |  |  |  |  |  |
| 1 | 0.999 (0.998-1.000) | 1.000 (0.999-1.000) | -0.001 (-0.002-0.001) | 0.404 | 0.999 (0.998-1.001) | 0.405 |
| 2 | 1.987 (1.983-1.992) | 1.989 (1.986-1.993) | -0.002 (-0.008-0.004) | 0.472 | 0.999 (0.996-1.002) | 0.472 |
| 3 | 2.966 (2.956-2.977) | 2.97 (2.962-2.979) | -0.004 (-0.017-0.010) | 0.580 | 0.999 (0.994-1.003) | 0.580 |
| 4 | 3.93 (3.913-3.947) | 3.935 (3.920-3.949) | -0.005 (-0.028-0.017) | 0.638 | 0.999 (0.993-1.004) | 0.638 |
| 5 | 4.864 (4.839-4.890) | 4.872 (4.850-4.894) | -0.008 (-0.042-0.025) | 0.638 | 0.998 (0.991-1.005) | 0.638 |
| 6 | 5.777 (5.742-5.812) | 5.79 (5.759-5.821) | -0.013 (-0.060-0.034) | 0.586 | 0.998 (0.990-1.006) | 0.586 |
| 7 | 6.671 (6.625-6.718) | 6.687 (6.646-6.728) | -0.016 (-0.078-0.046) | 0.619 | 0.998 (0.988-1.007) | 0.619 |
| 8 | 7.543 (7.485-7.602) | 7.568 (7.517-7.620) | -0.025 (-0.103-0.053) | 0.528 | 0.997 (0.986-1.007) | 0.528 |
| Liver resection subgroup |  |  |  |  |  |  |
| 1 | 0.999 (0.999-1.000) | 1.000 (1.000-1.000) | 0.000 (-0.001-0.000) | 0.321 | 1.000 (0.999-1.000) | 0.321 |
| 2 | 1.989 (1.984-1.993) | 1.991 (1.987-1.995) | -0.002 (-0.008-0.003) | 0.403 | 0.999 (0.996-1.002) | 0.403 |
| 3 | 2.969 (2.959-2.979) | 2.973 (2.964-2.981) | -0.004 (-0.017-0.009) | 0.592 | 0.999 (0.994-1.003) | 0.592 |
| 4 | 3.933 (3.917-3.950) | 3.938 (3.923-3.952) | -0.004 (-0.027-0.018) | 0.696 | 0.999 (0.993-1.005) | 0.696 |
| 5 | 4.869 (4.844-4.894) | 4.876 (4.854-4.899) | -0.007 (-0.041-0.026) | 0.673 | 0.999 (0.992-1.005) | 0.673 |
| 6 | 5.783 (5.747-5.818) | 5.797 (5.765-5.828) | -0.014 (-0.061-0.034) | 0.569 | 0.998 (0.989-1.006) | 0.569 |
| 7 | 6.677 (6.630-6.724) | 6.695 (6.653-6.737) | -0.018 (-0.081-0.045) | 0.577 | 0.997 (0.988-1.007) | 0.577 |
| 8 | 7.550 (7.490-7.609) | 7.580 (7.527-7.633) | -0.030 (-0.11-0.049) | 0.454 | 0.996 (0.986-1.007) | 0.454 |

TDF, tenofovir; ETV, entecavir; CI, confidence interval.

**Supplementary Table S4.** restricted mean survival time analysis of death rate in patients treated with TDF vs. ETV.

| Follow-up year | RMST | | RMST difference (95% CI) | *P*-value | RMST ratio (95% CI) | *P*-value |
| --- | --- | --- | --- | --- | --- | --- |
|  | TDF | ETV |  |  |  |  |
| Overall cohort |  |  |  |  |  |  |
| 1 | 1.000 (0.999-1.000) | 1.000 (1.000-1.000) | 0.000 (-0.001-0.000) | 0.317 | 1.000 (0.999-1.000) | 0.317 |
| 2 | 1.999 (1.997-2.001) | 1.999 (1.998-2.000) | 0.000 (-0.002-0.002) | 0.861 | 1.000 (0.999-1.001) | 0.861 |
| 3 | 2.999 (2.996-3.001) | 2.998 (2.996-3.000) | 0.001 (-0.003-0.004) | 0.704 | 1.000 (0.999-1.001) | 0.704 |
| 4 | 3.996 (3.991-4.000) | 3.994 (3.990-3.998) | 0.002 (-0.004-0.008) | 0.597 | 1.000 (0.999-1.002) | 0.597 |
| 5 | 4.991 (4.984-4.997) | 4.987 (4.980-4.994) | 0.004 (-0.006-0.013) | 0.427 | 1.001 (0.999-1.003) | 0.427 |
| 6 | 5.982 (5.973-5.992) | 5.976 (5.966-5.986) | 0.006 (-0.008-0.02) | 0.417 | 1.001 (0.999-1.003) | 0.417 |
| 7 | 6.971 (6.958-6.985) | 6.959 (6.945-6.973) | 0.012 (-0.008-0.031) | 0.230 | 1.002 (0.999-1.004) | 0.230 |
| 8 | 7.957 (7.940-7.975) | 7.935 (7.916-7.954) | 0.022 (-0.004-0.048) | 0.092 | 1.003 (1.000-1.006) | 0.092 |
| Liver resection subgroup |  |  |  |  |  |  |
| 1 | 1.000 (0.999-1.000) | 1.000 (1.000-1.000) | 0.000 (-0.001-0.000) | 0.317 | 1.000 (0.999-1.000) | 0.317 |
| 2 | 1.999 (1.997-2.001) | 1.999 (1.998-2.000) | 0.000 (-0.002-0.002) | 0.875 | 1.000 (0.999-1.001) | 0.875 |
| 3 | 2.998 (2.996-3.001) | 2.998 (2.995-3.000) | 0.001 (-0.003-0.005) | 0.686 | 1.000 (0.999-1.002) | 0.686 |
| 4 | 3.995 (3.991-4.000) | 3.993 (3.989-3.998) | 0.002 (-0.004-0.008) | 0.567 | 1.000 (0.999-1.002) | 0.567 |
| 5 | 4.990 (4.983-4.997) | 4.986 (4.979-4.993) | 0.004 (-0.006-0.014) | 0.393 | 1.001 (0.999-1.003) | 0.393 |
| 6 | 5.981 (5.971-5.991) | 5.974 (5.964-5.985) | 0.007 (-0.008-0.022) | 0.375 | 1.001 (0.999-1.004) | 0.375 |
| 7 | 6.970 (6.956-6.984) | 6.956 (6.941-6.971) | 0.014 (-0.007-0.034) | 0.197 | 1.002 (0.999-1.005) | 0.197 |
| 8 | 7.955 (7.936-7.973) | 7.930 (7.909-7.95) | 0.025 (-0.002-0.053) | 0.073 | 1.003 (1-1.007) | 0.073 |

TDF, tenofovir; ETV, entecavir; CI, confidence interval.

**Supplementary Figure S1.** Reconstructed survival curves.

**He et al 2023: RFS**


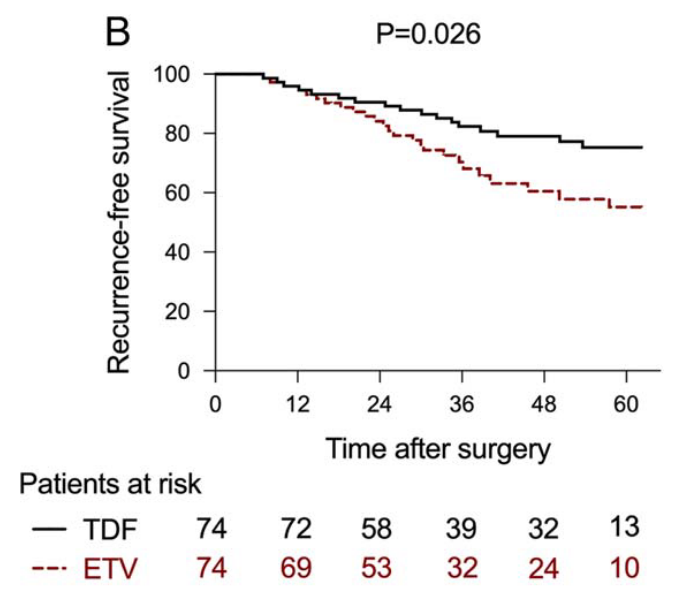
Original


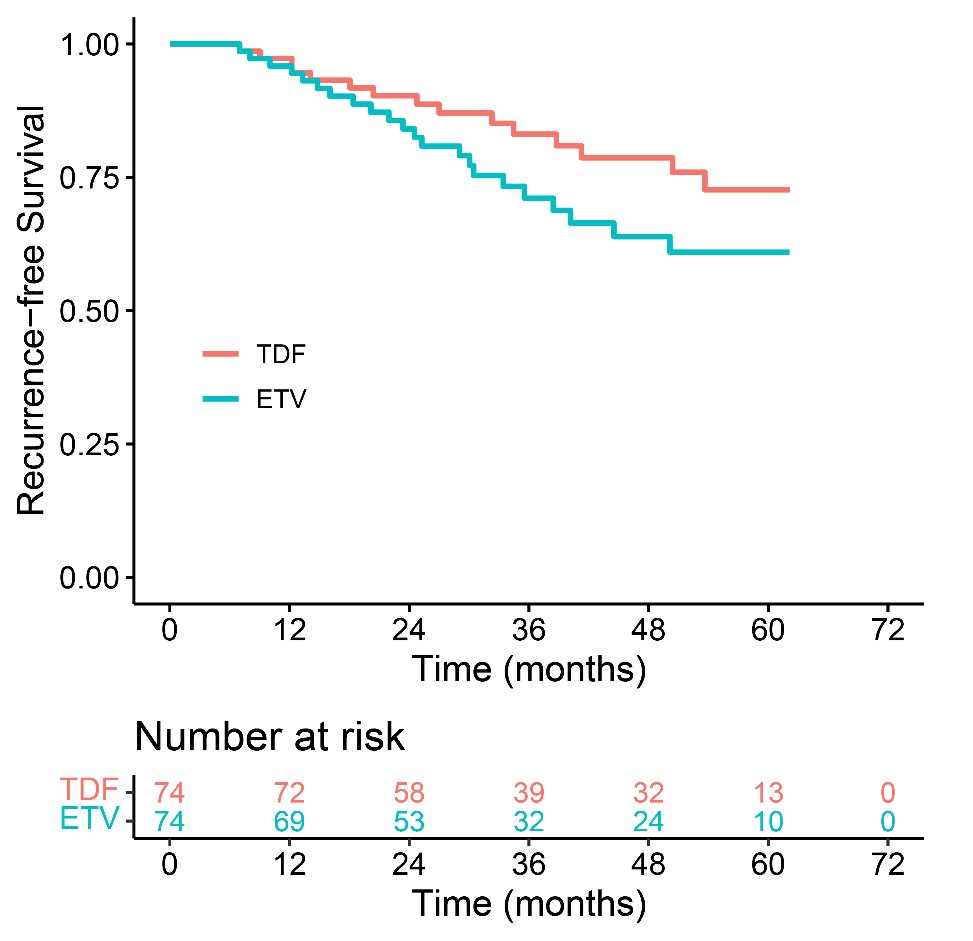
Reconstructed

**He et al 2023: OS**

Original


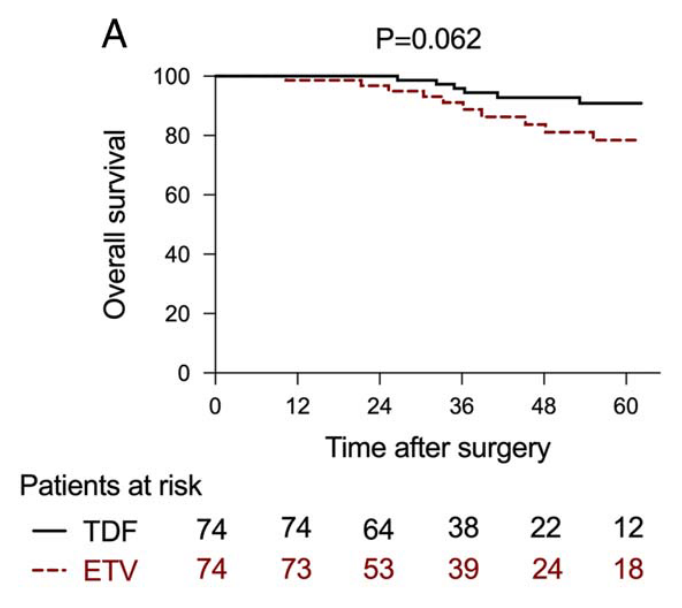


Reconstructed


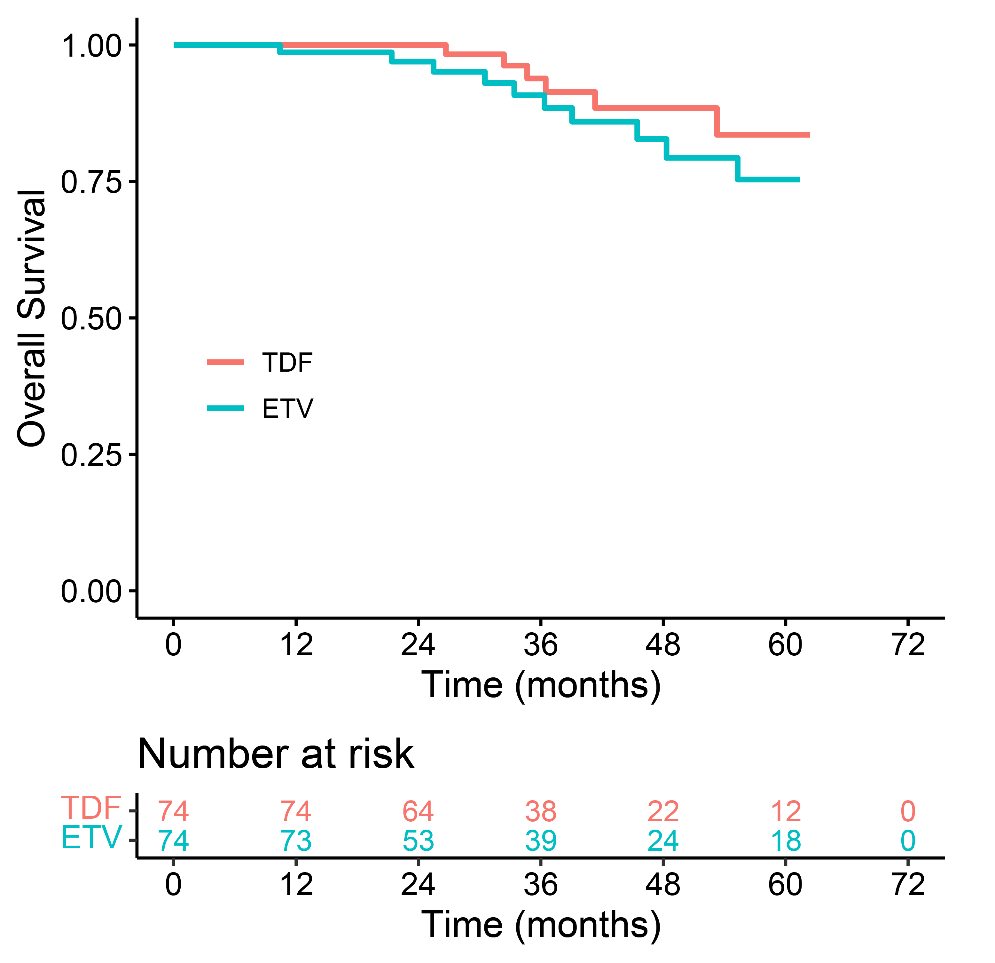
**Hu et al 2022: RFS**


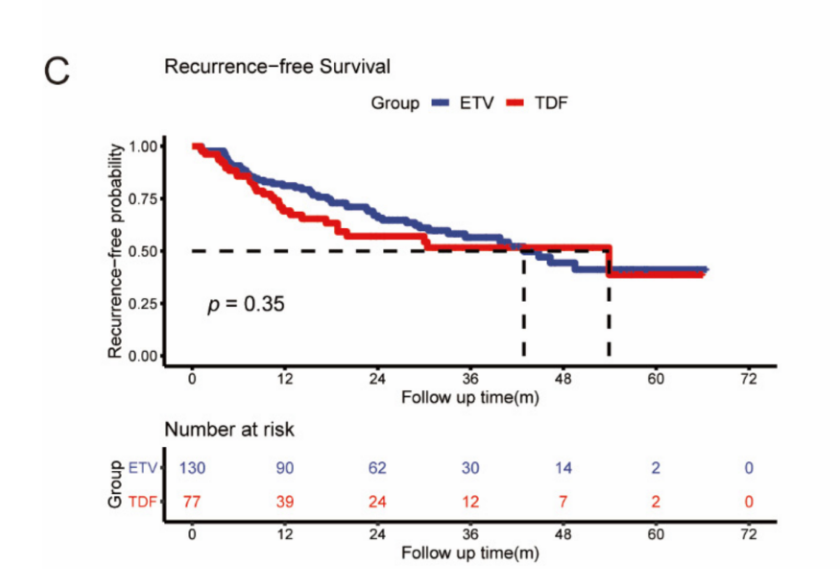
Original


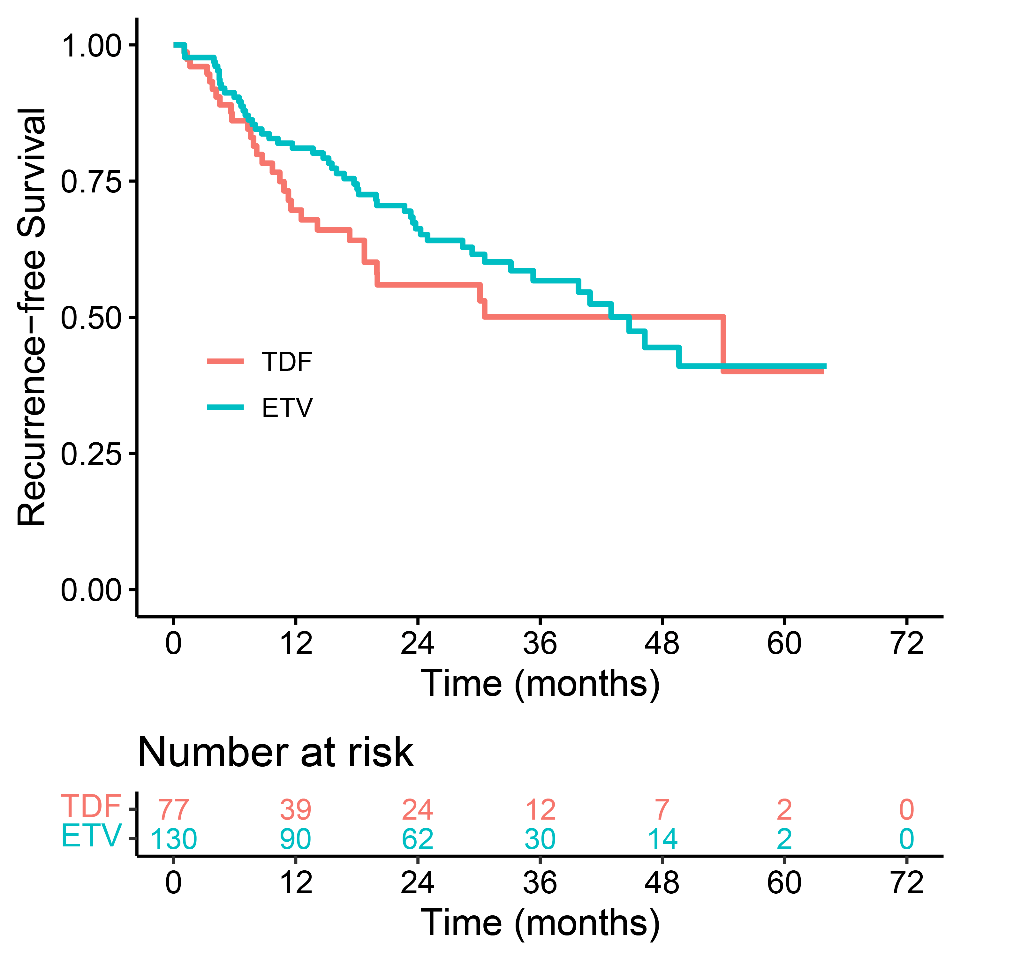
Reconstructed

**Hu et al 2022: OS**


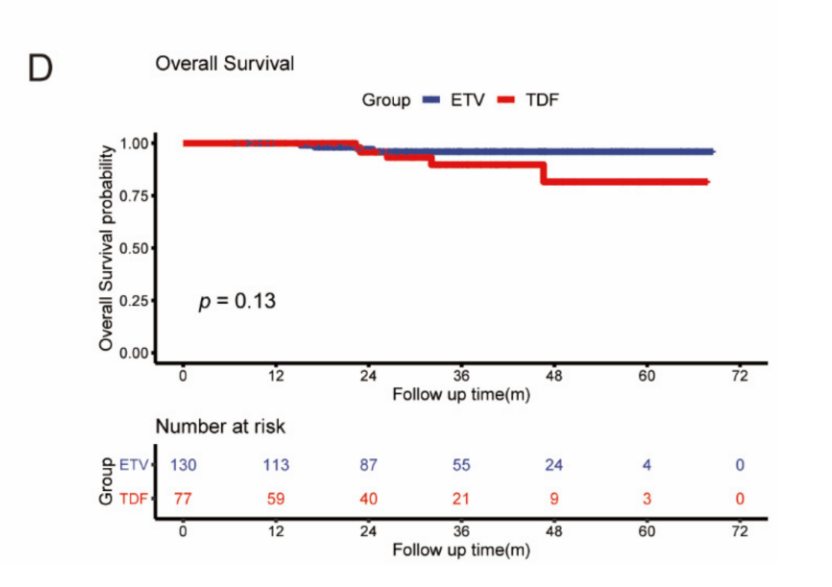
Original


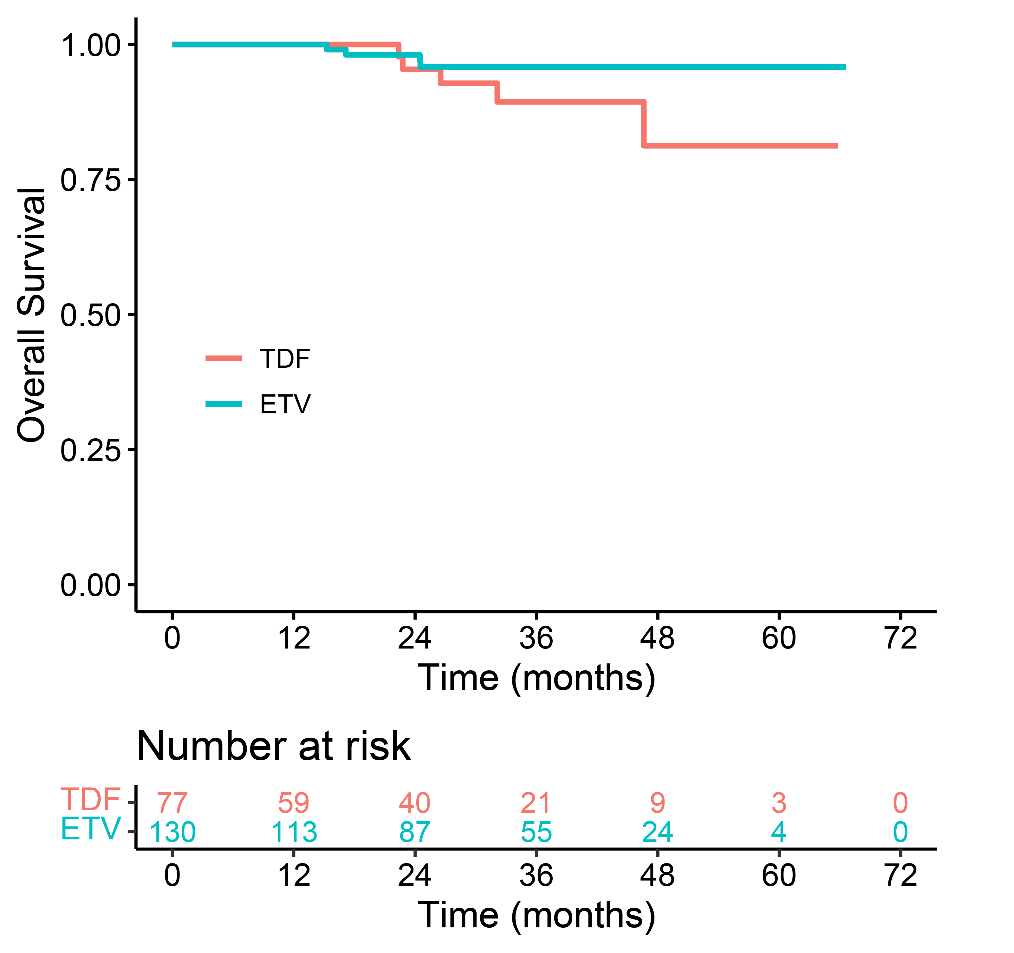
Reconstructed

**Qi et al 2021: RFS**


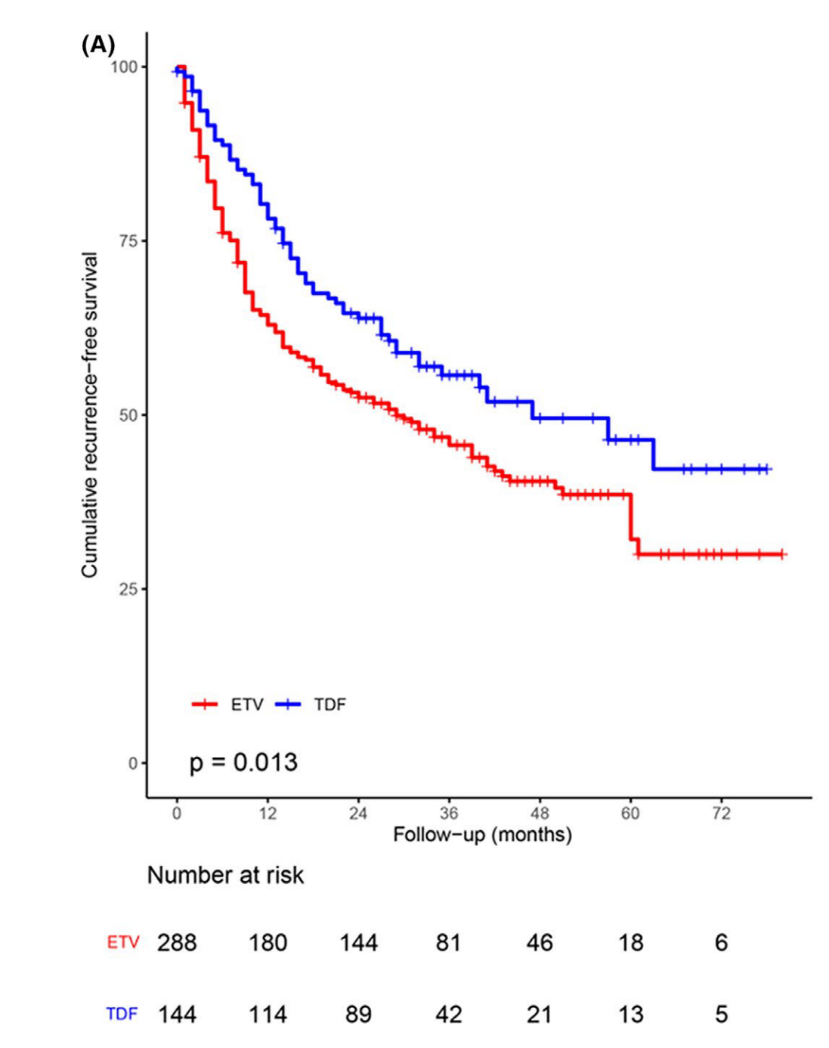
Original


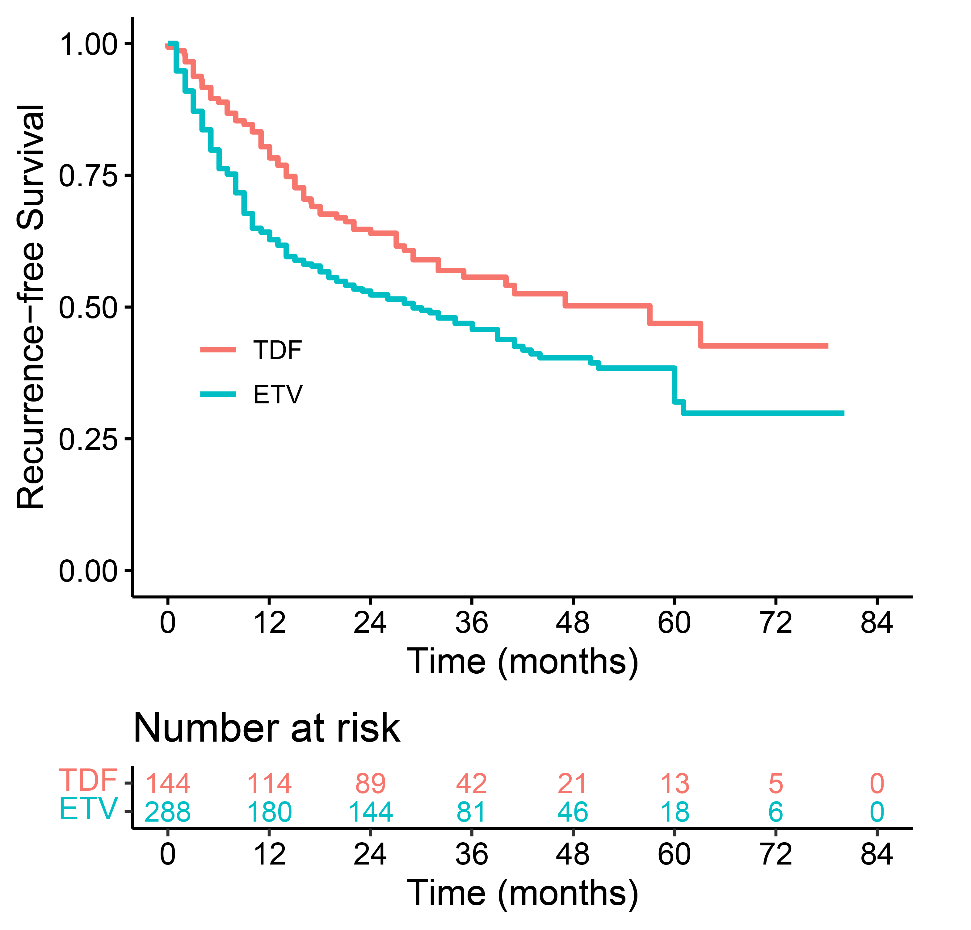
Reconstructed

**Qi et al 2021: OS**

**
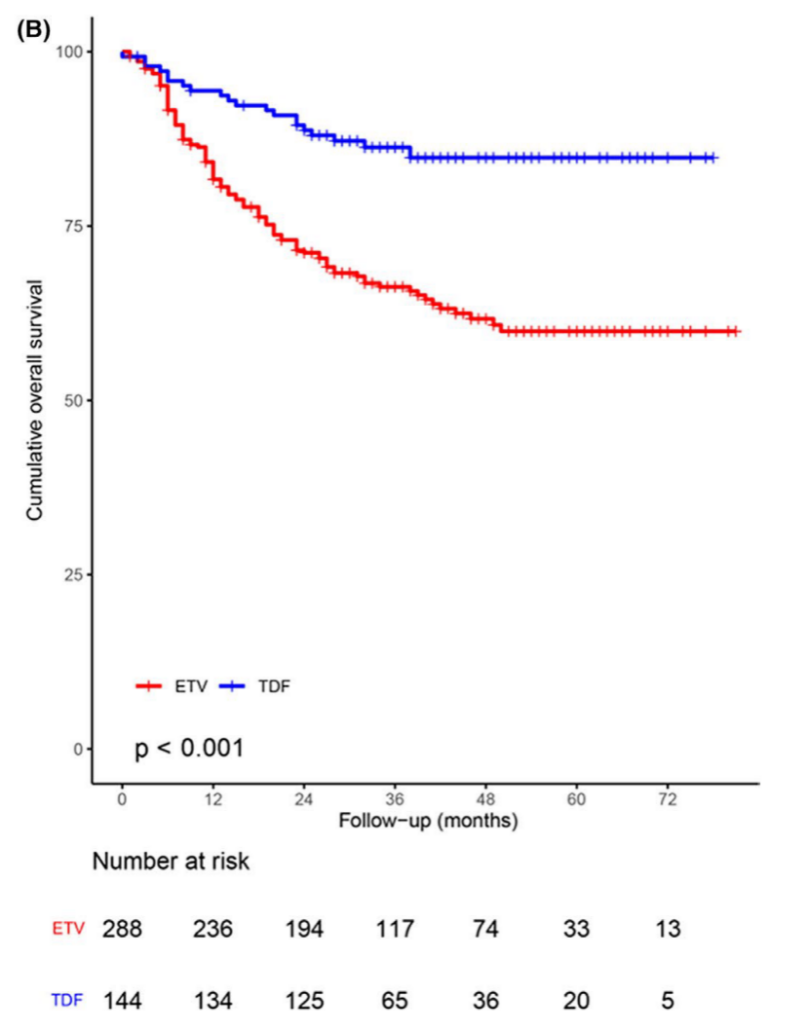
**Original

Reconstructed


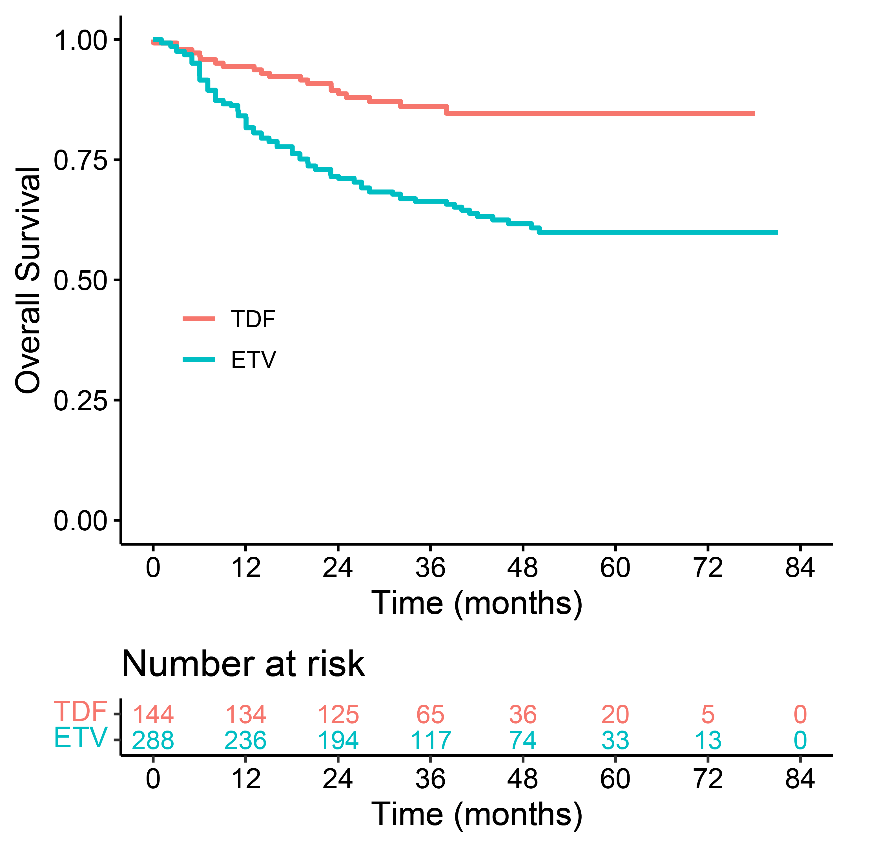
**Tsai et al 2022: RFS**


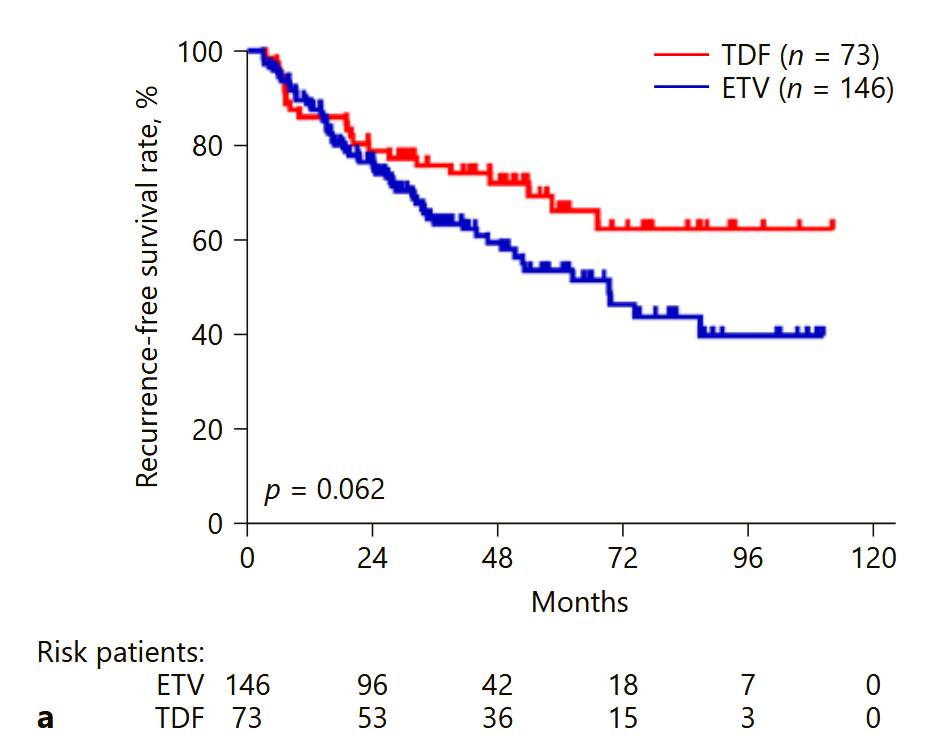
Original


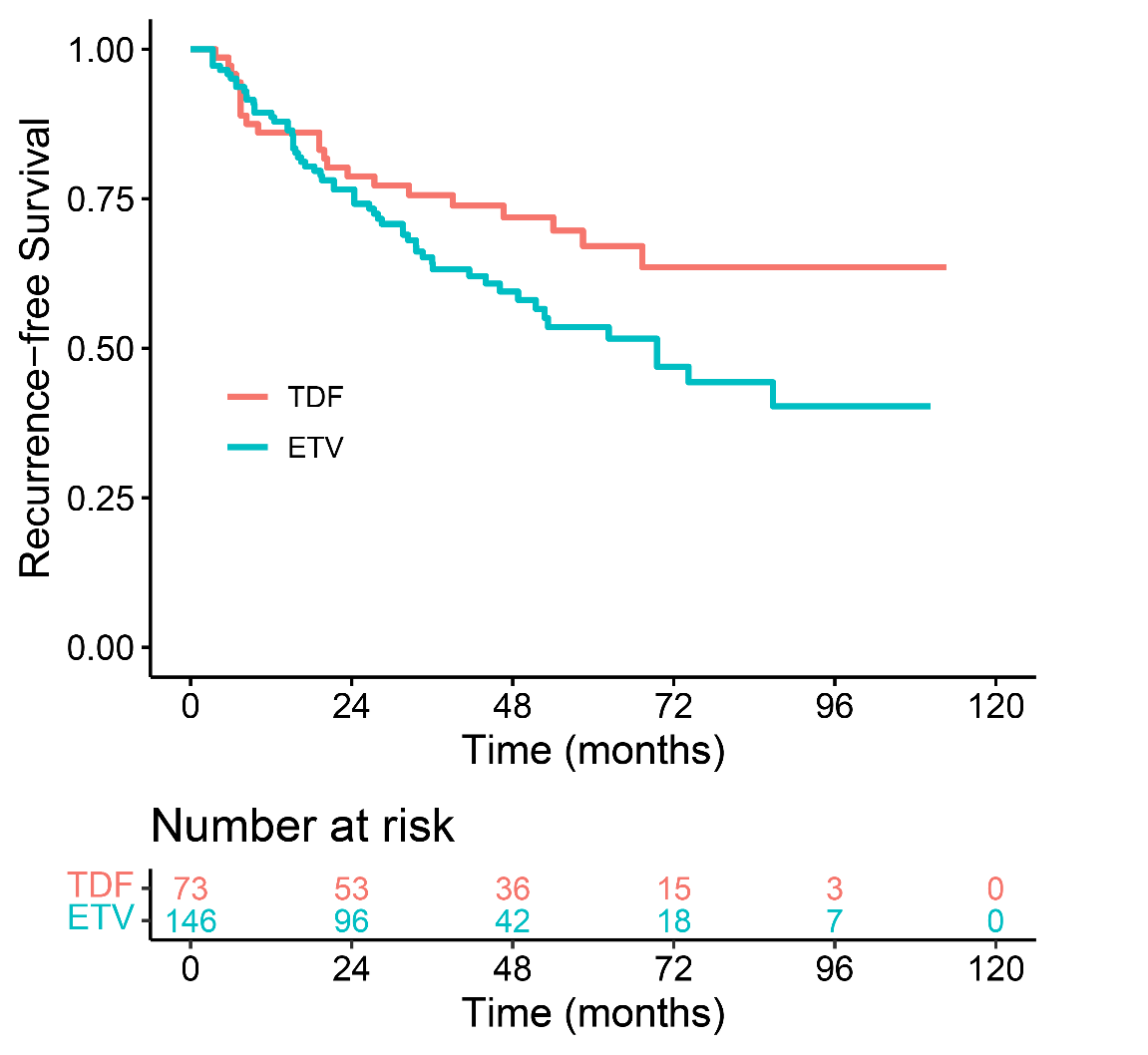
Reconstructed

**Tsai et al 2022: OS**


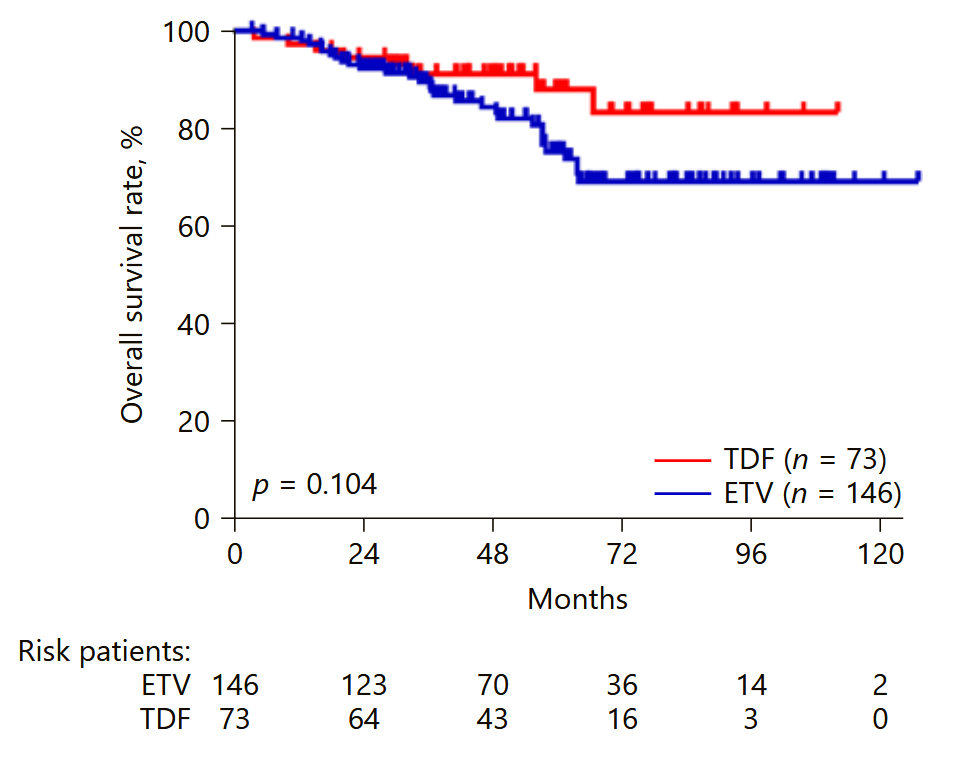
Original


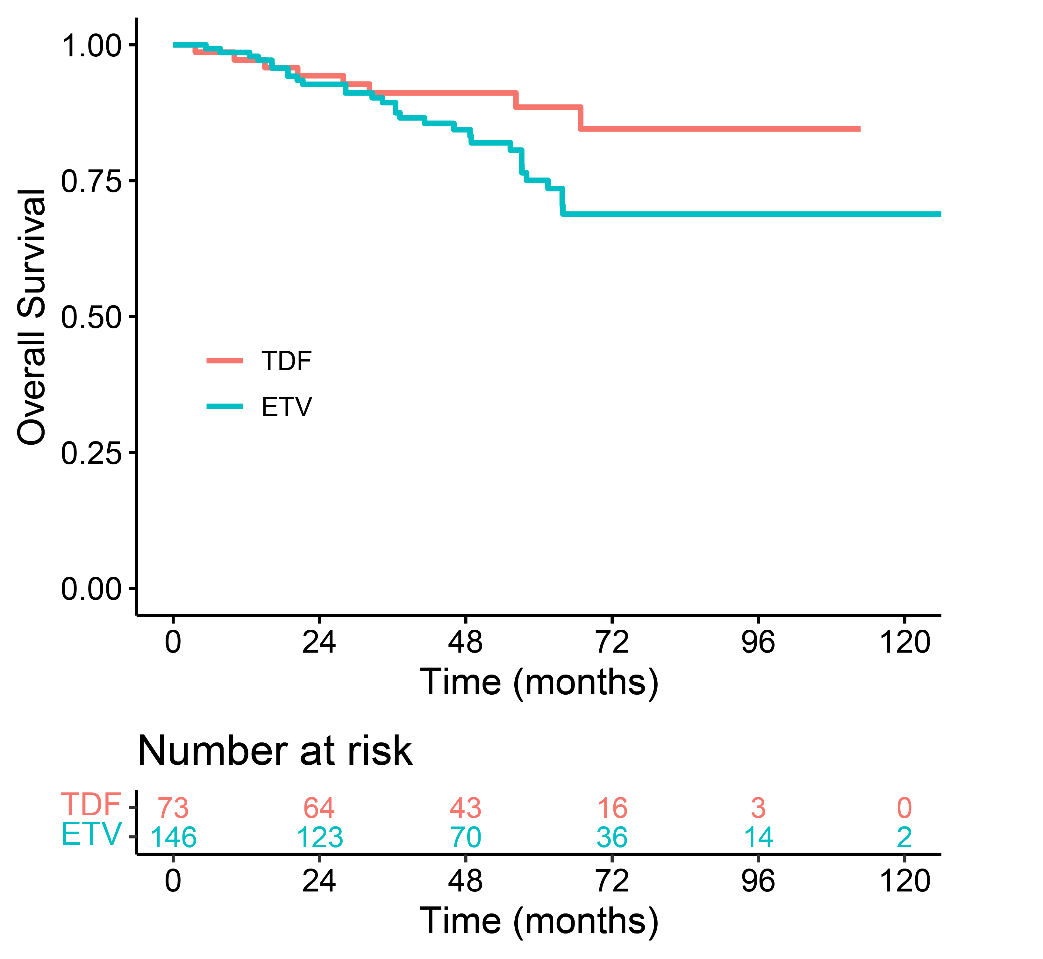
Reconstructed

**Wang et al 2022: RFS**


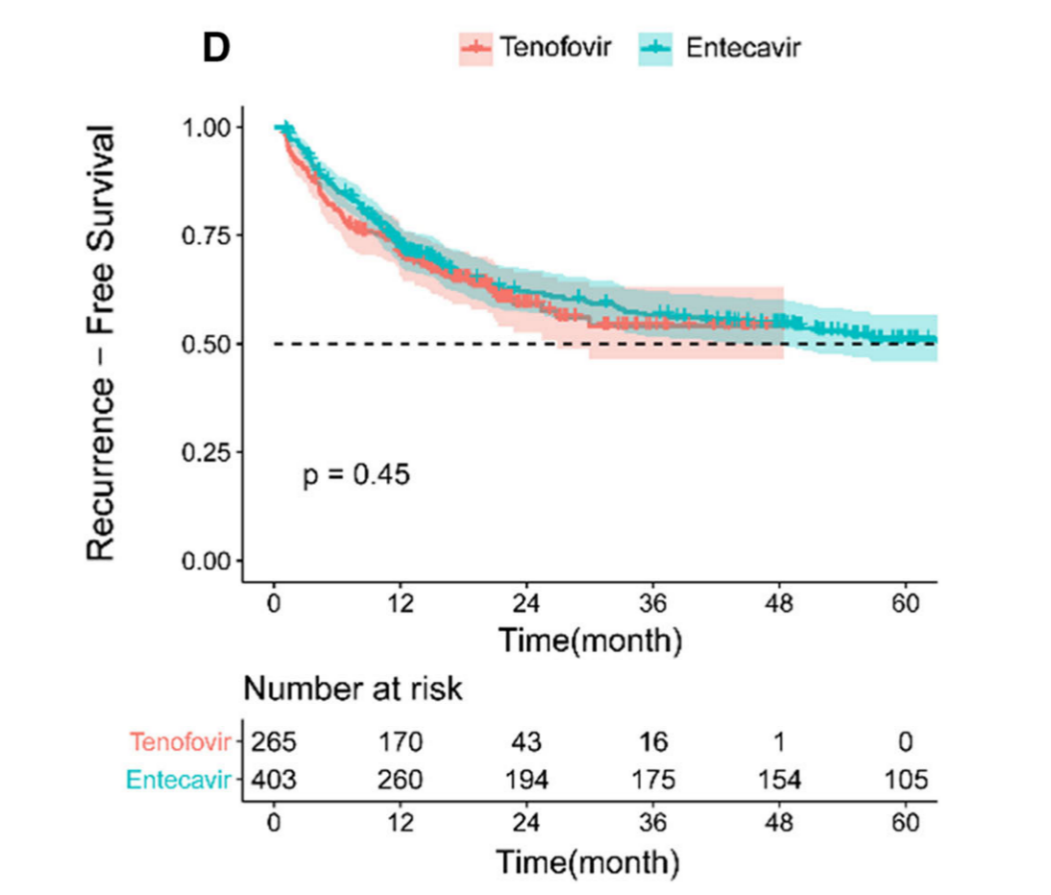
Original


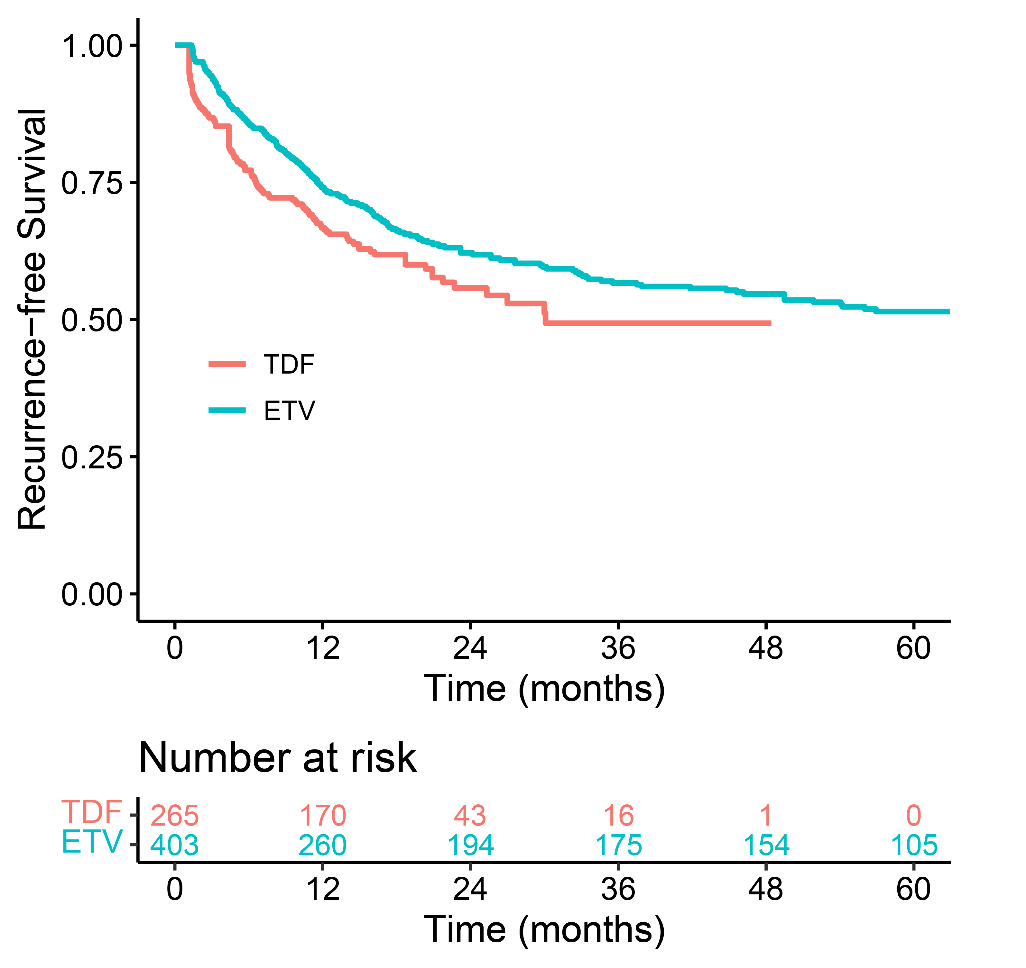
Reconstructed

**Wang et al 2022: OS**


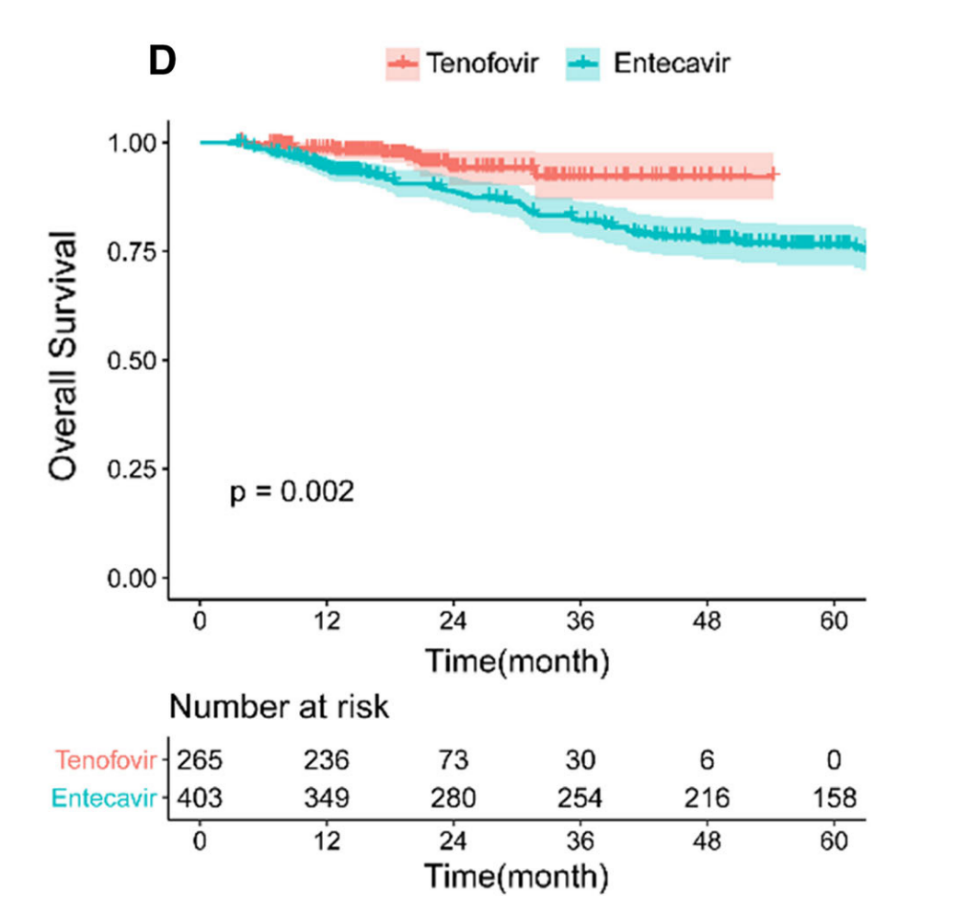
Original


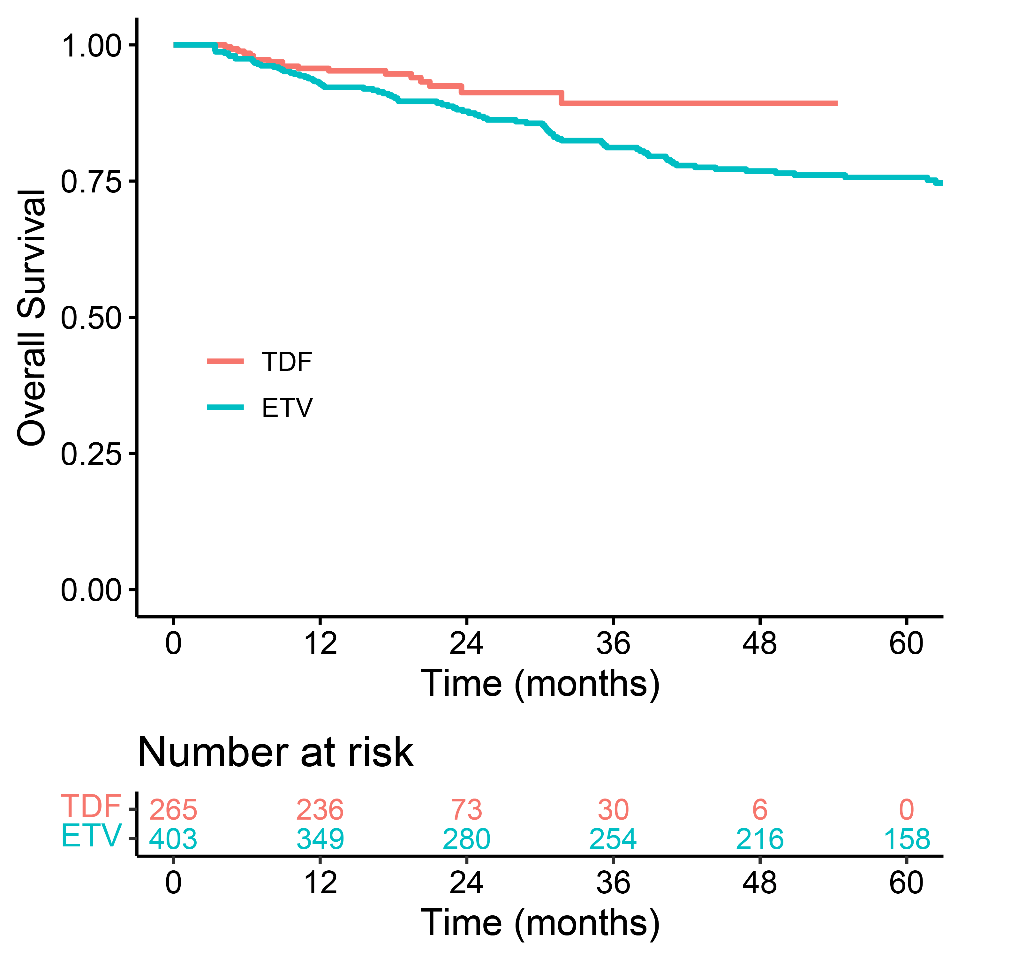
Reconstructed

**Yang et al 2023: RFS**


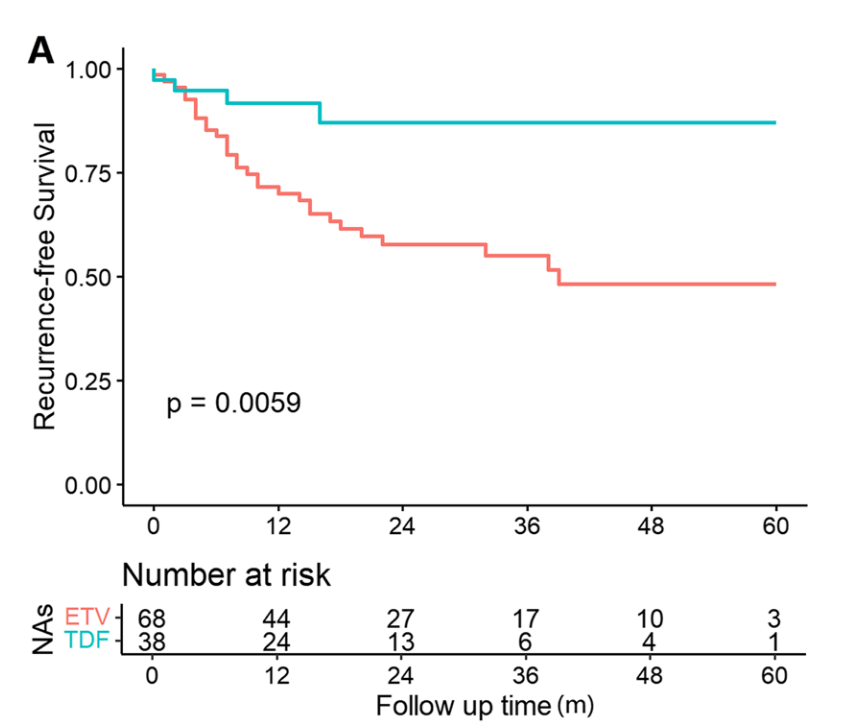
Original


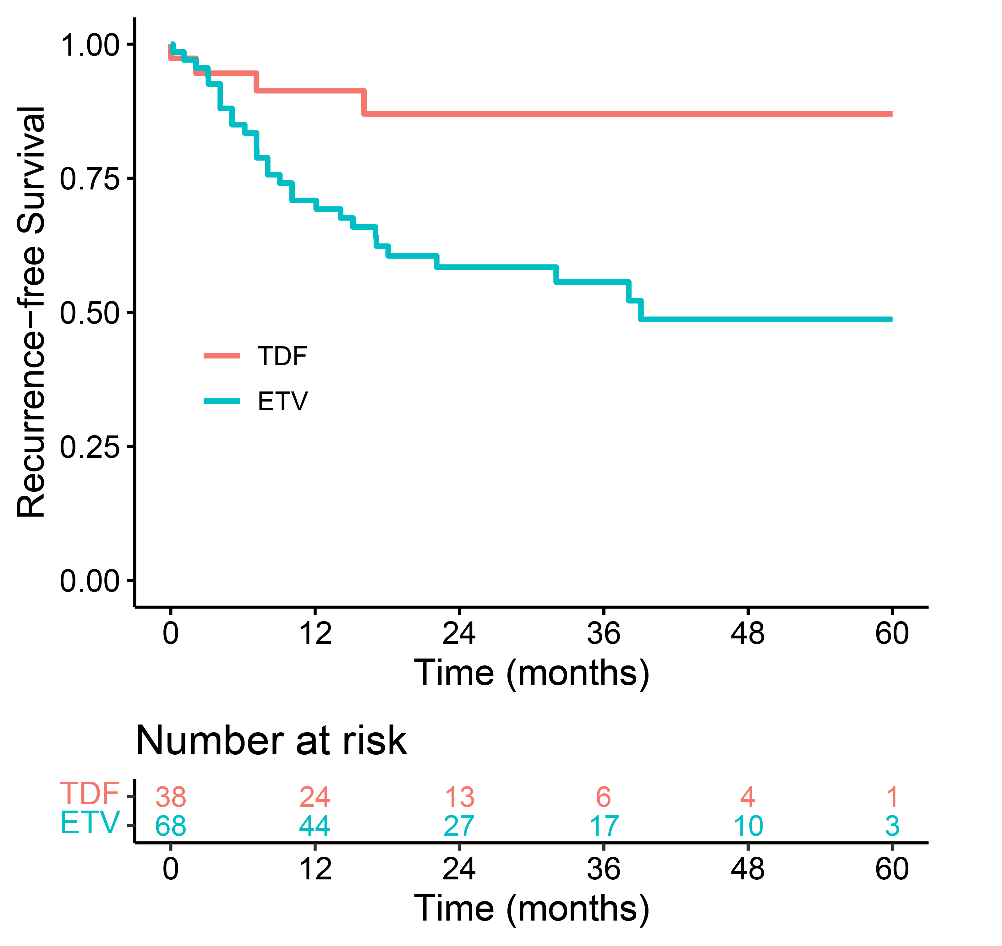
Reconstructed

**Yang et al 2023: OS**


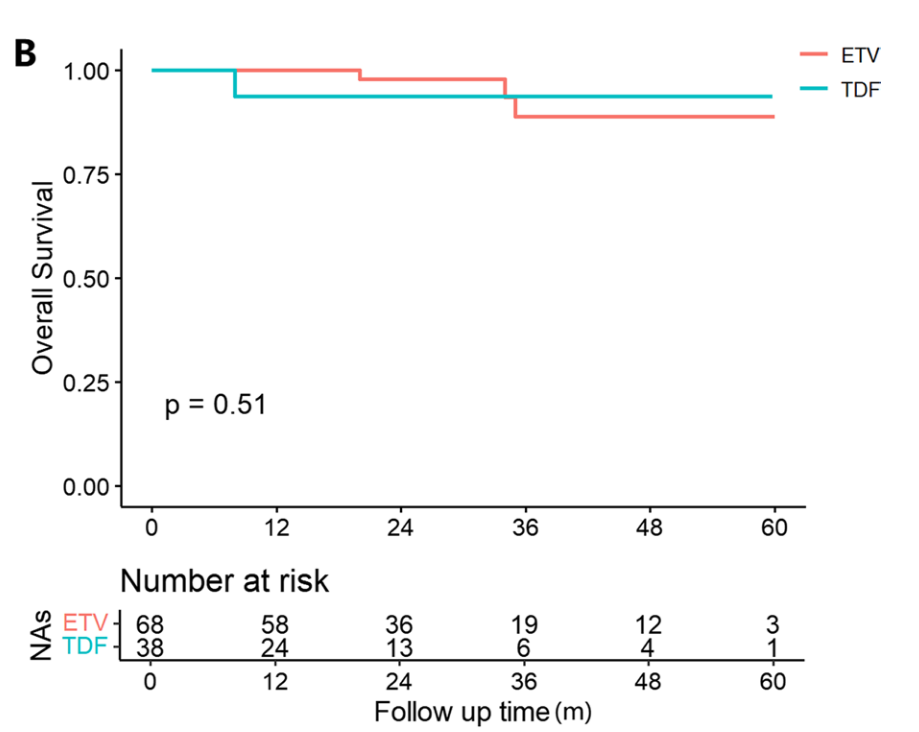
Original


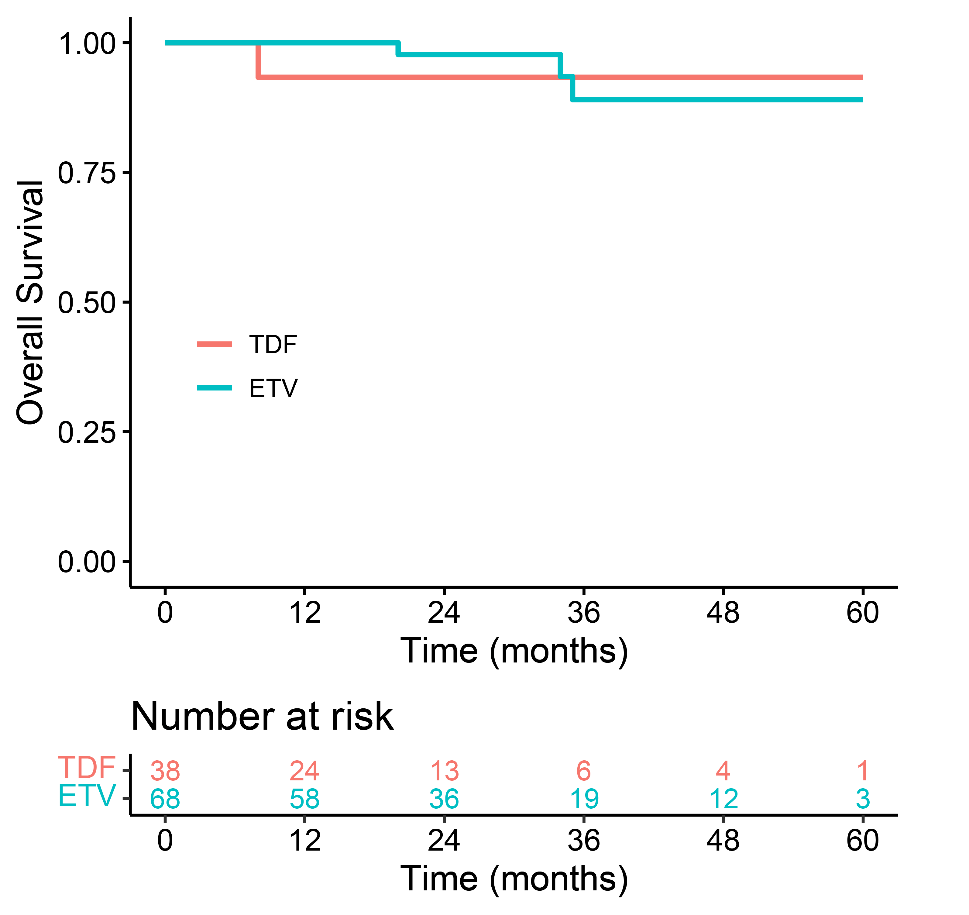
Reconstructed

**Yun et al 2022: RFS**


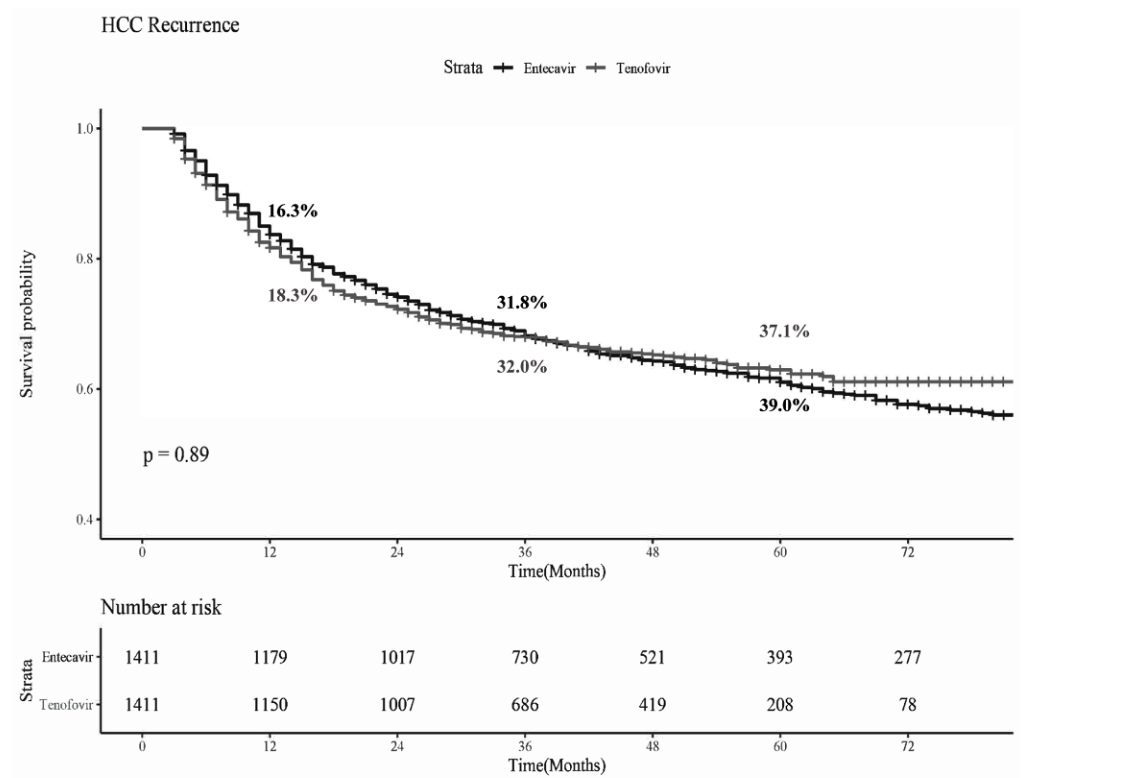
Original


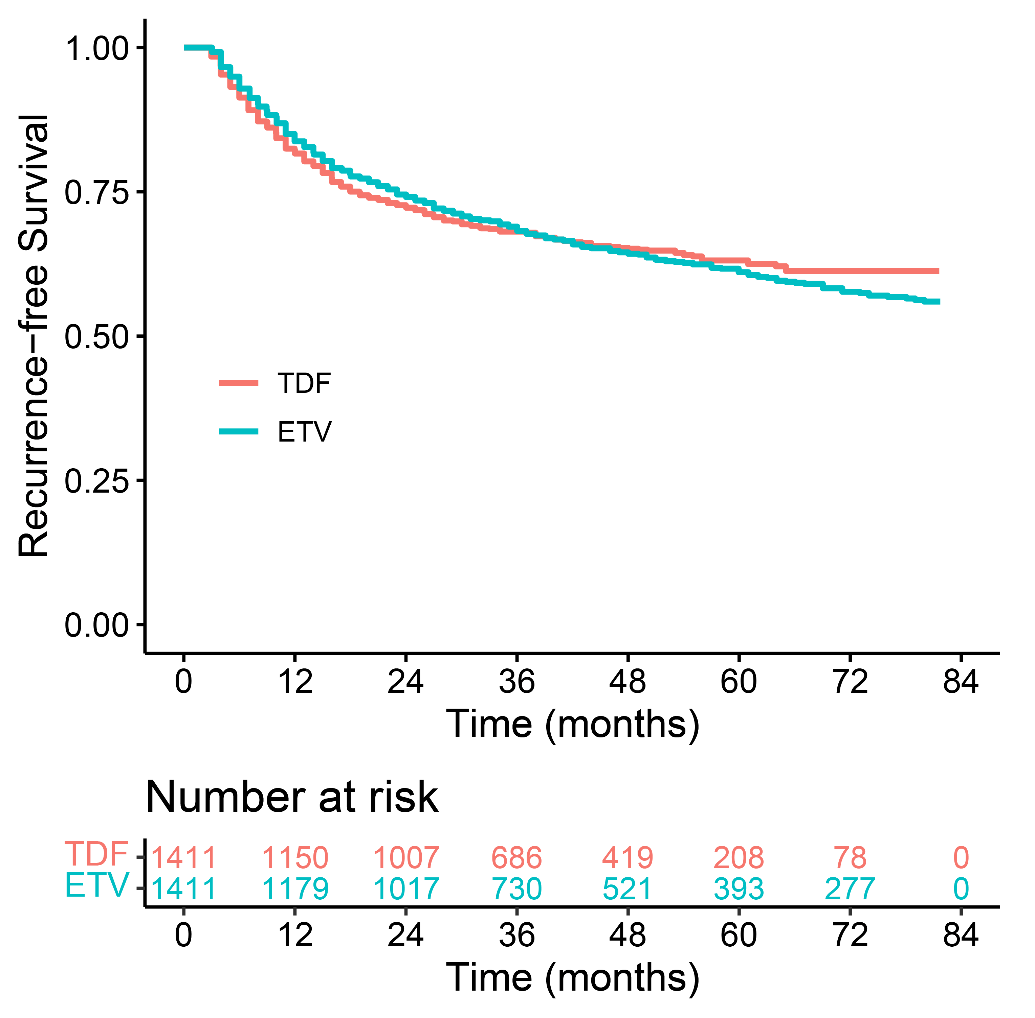
Reconstructed

**Yun et al 2022: OS**


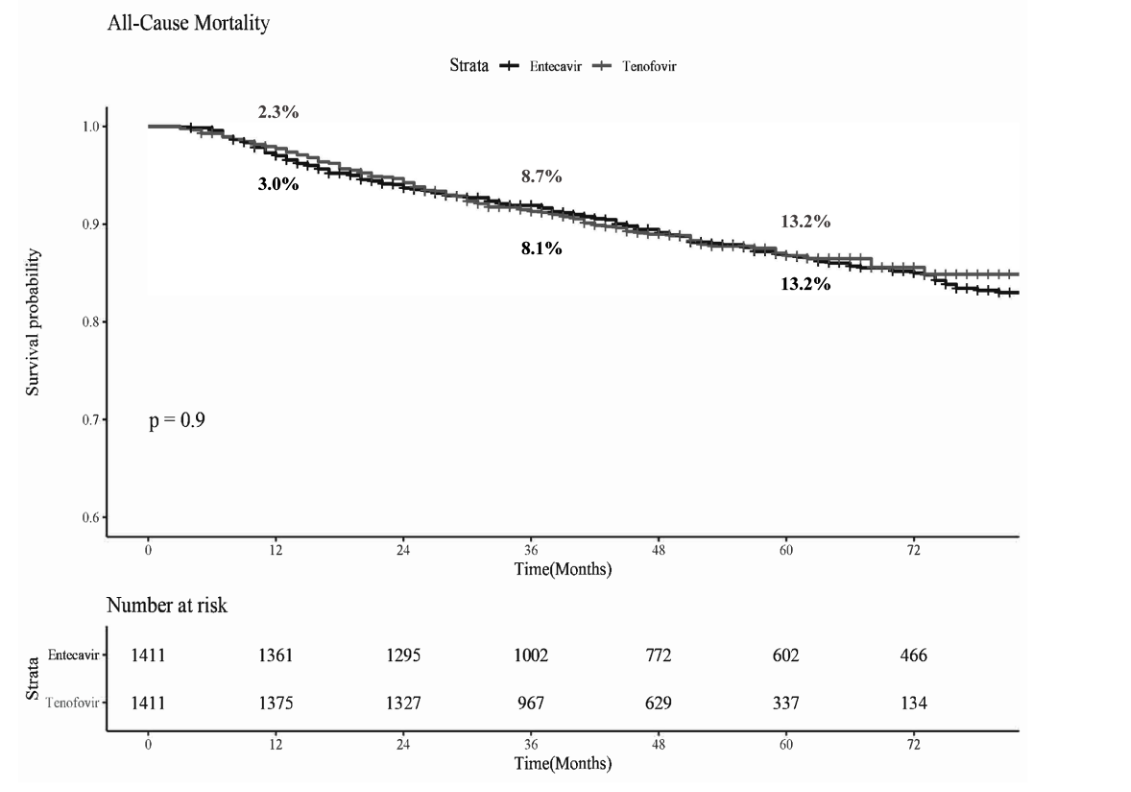
Original


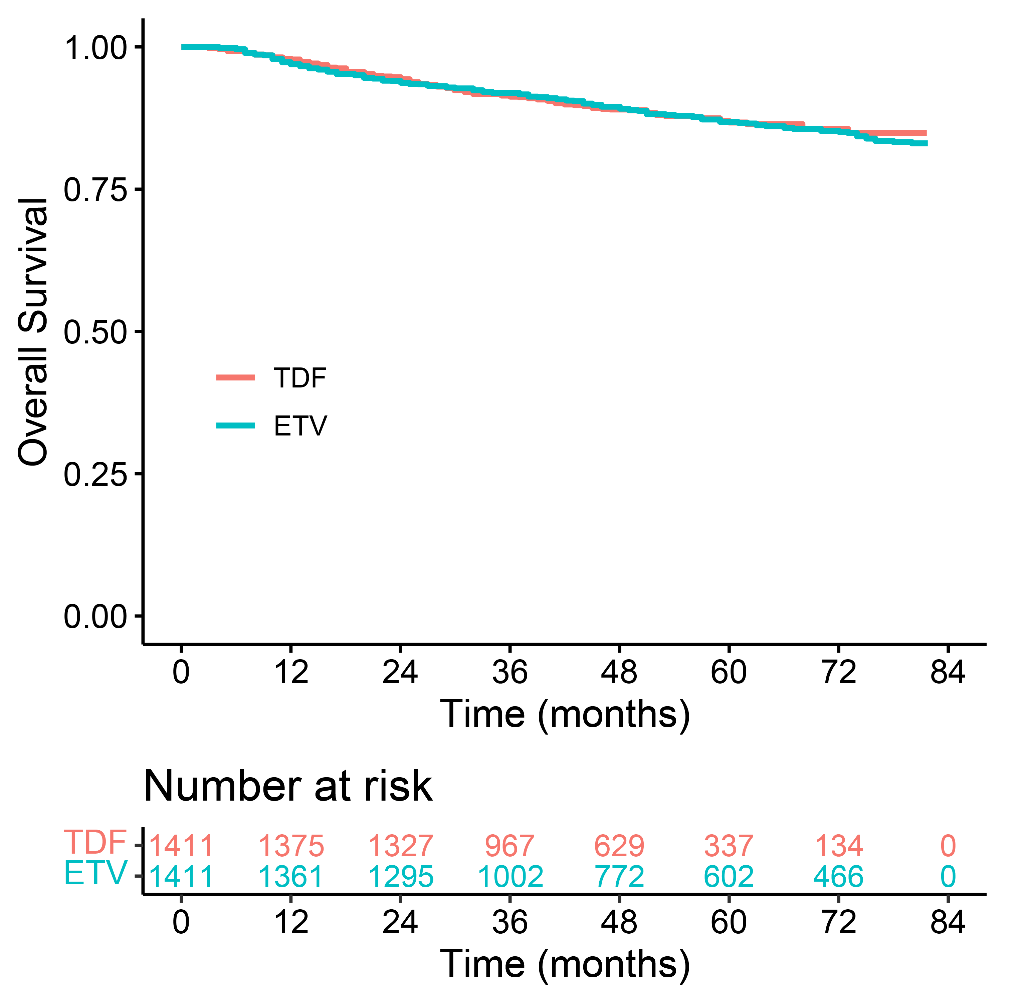
Reconstructed

**Supplementary Figure S2.** Assessment of publication bias.

1. Funnel plot for analyzing RFS in overall cohort


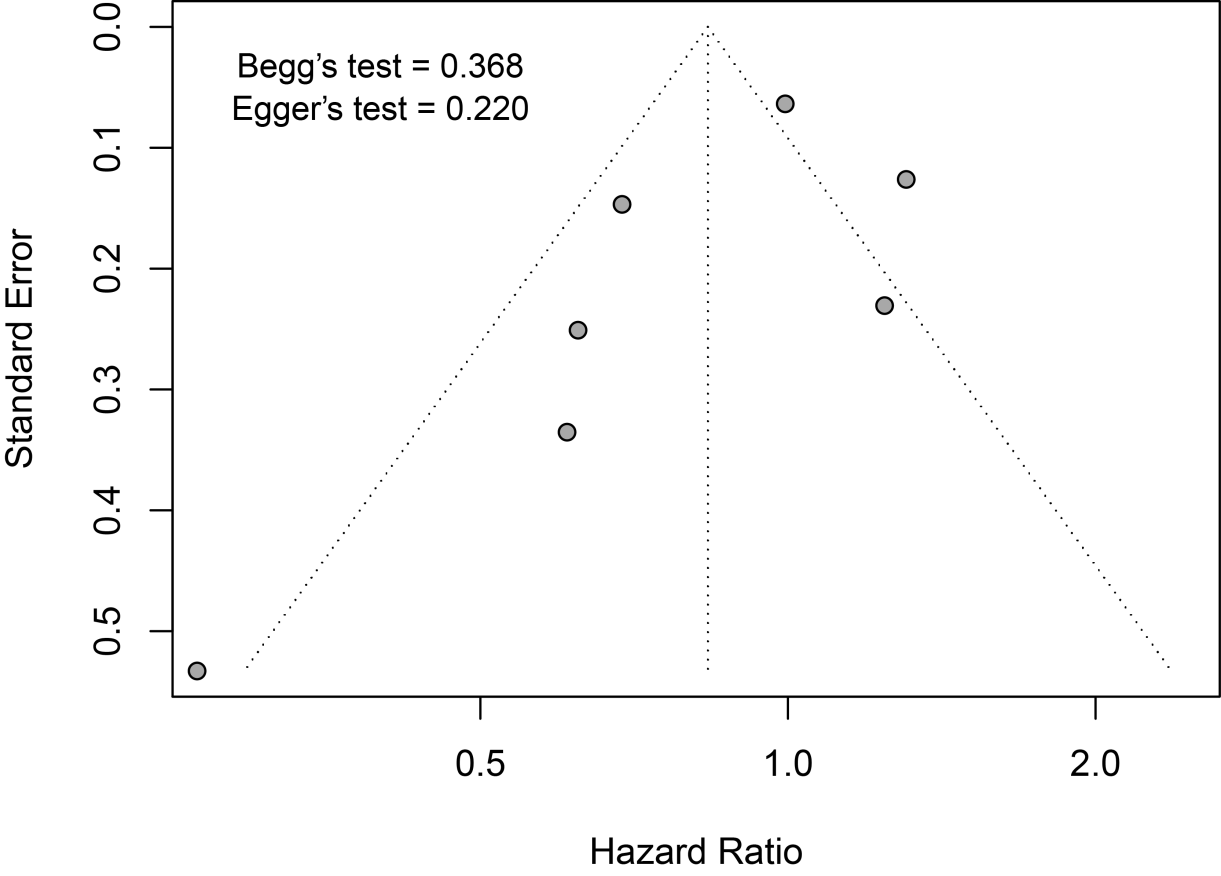


1. Funnel plot for analyzing OS in overall cohort


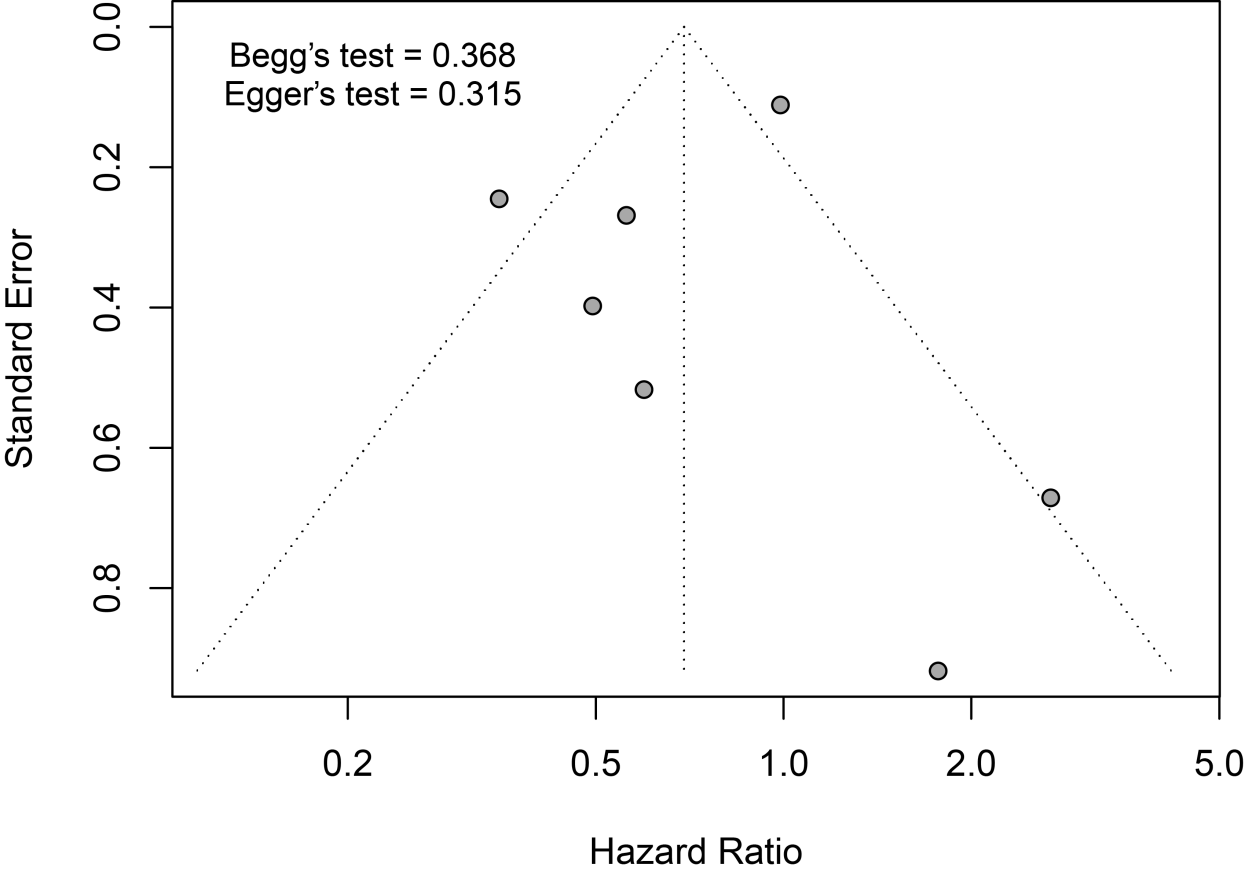


1. Funnel plot for analyzing RFS in resection subgroup


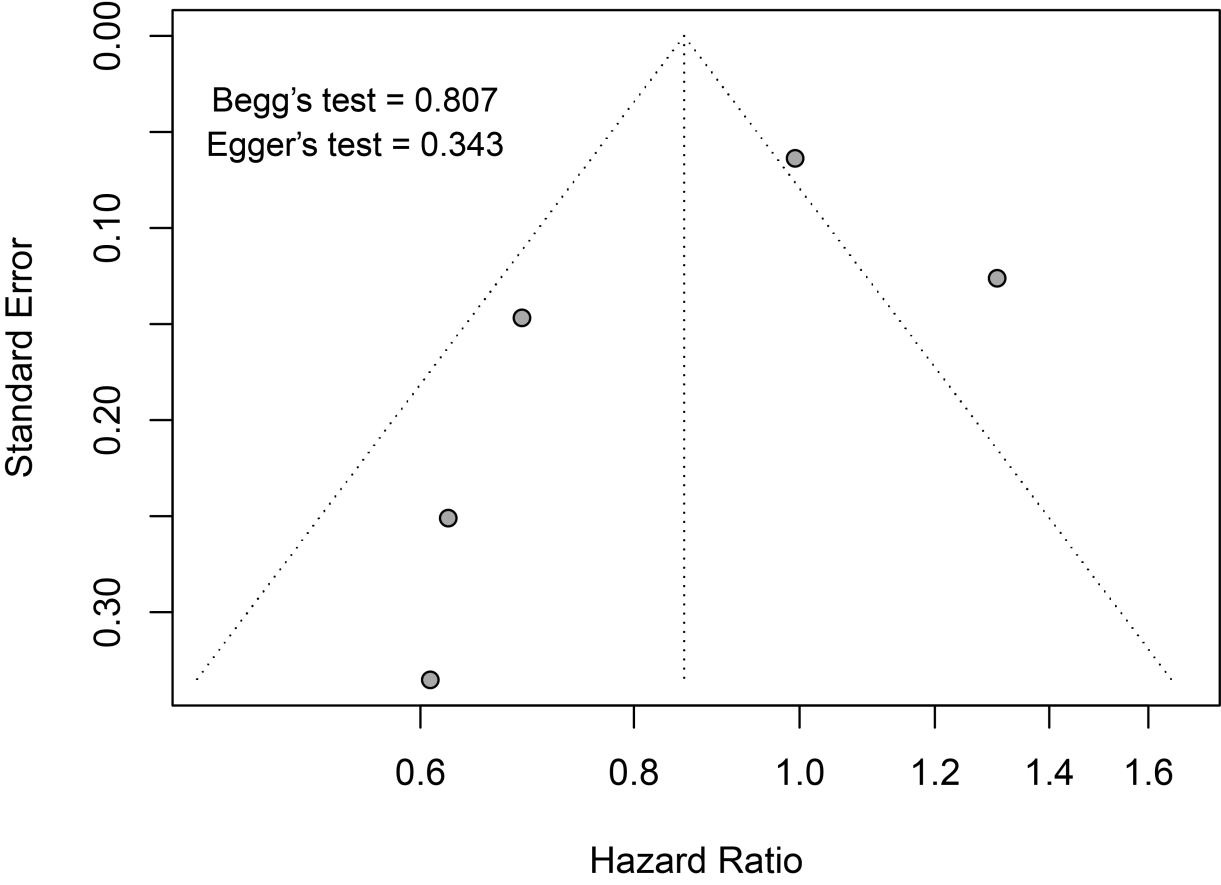


1. Funnel plot for analyzing OS in resection subgroup


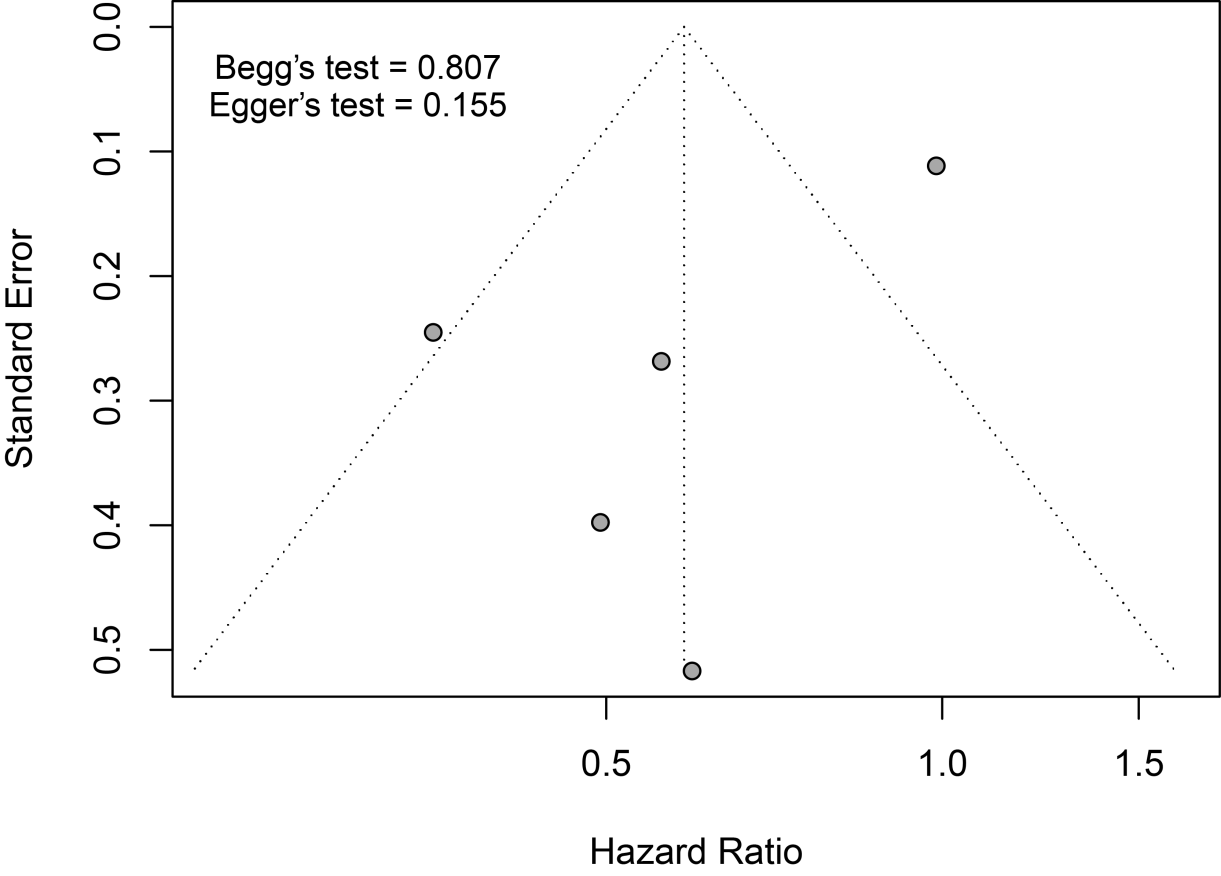


**Supplementary Figure S3.** Sensitivity analysis.

A. Sensitivity analysis for RFS in overall cohort

**
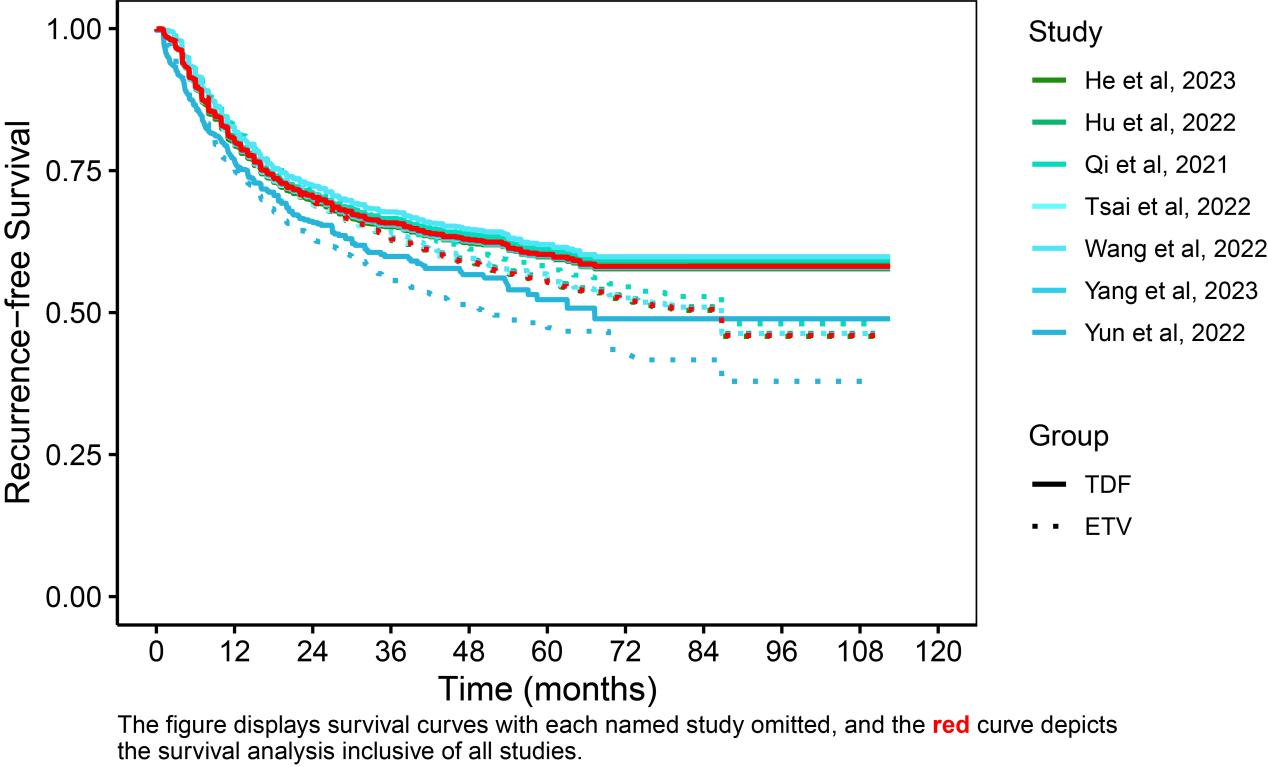
**

1. Sensitivity analysis for OS in overall cohort

**
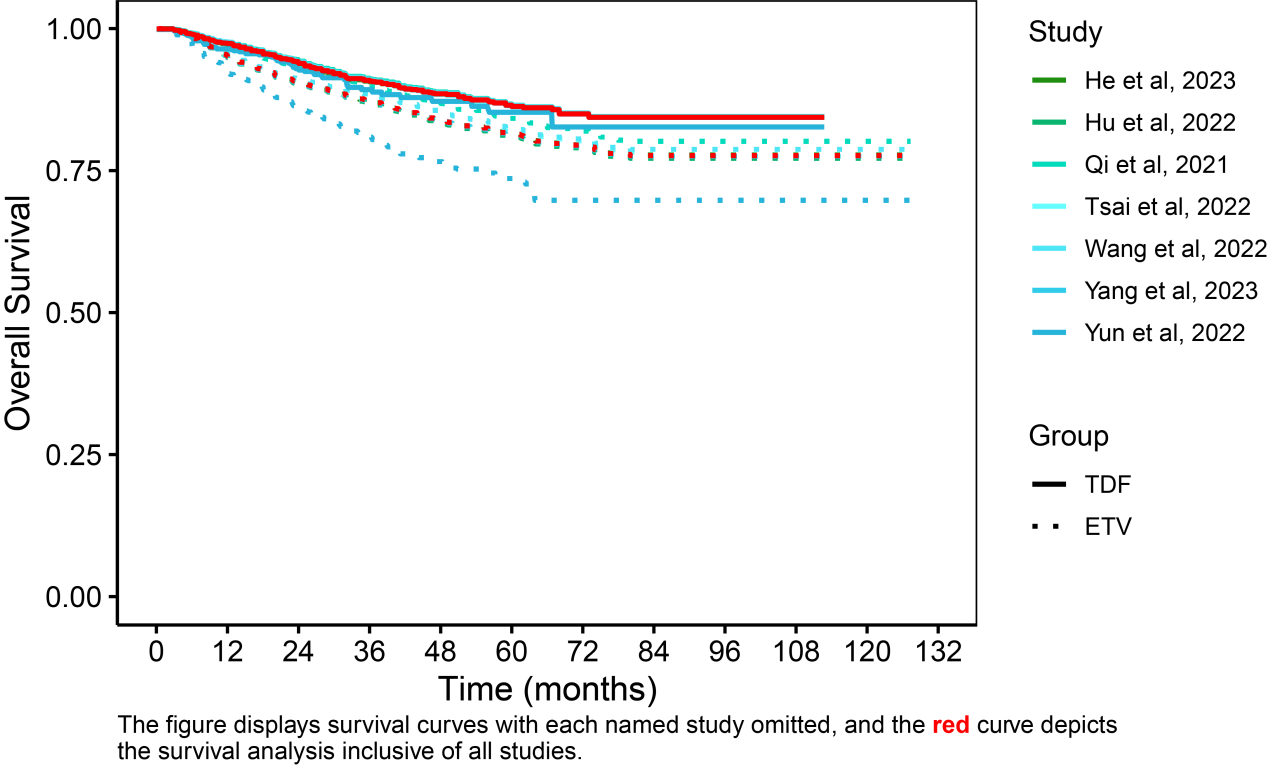
**

C. Sensitivity analysis for RFS in resection subgroup

**
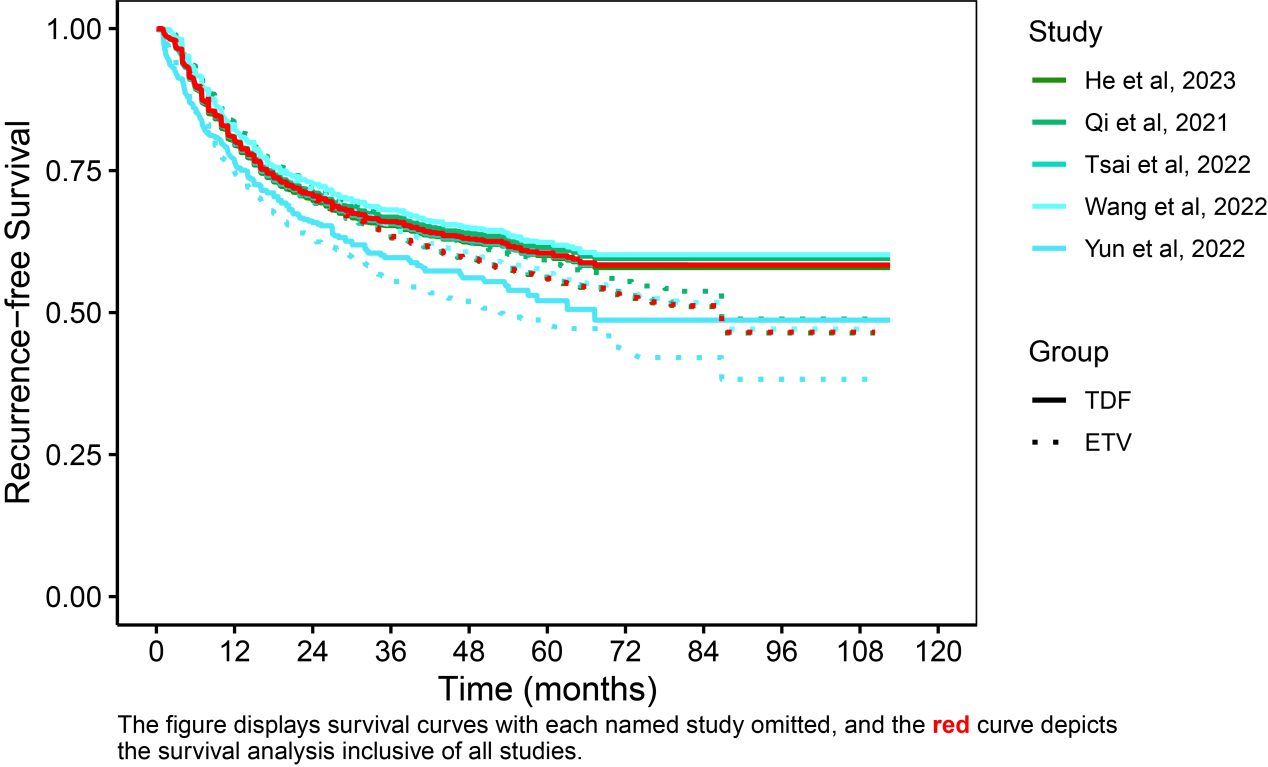
**

D. Sensitivity analysis for OS in resection subgroup

**
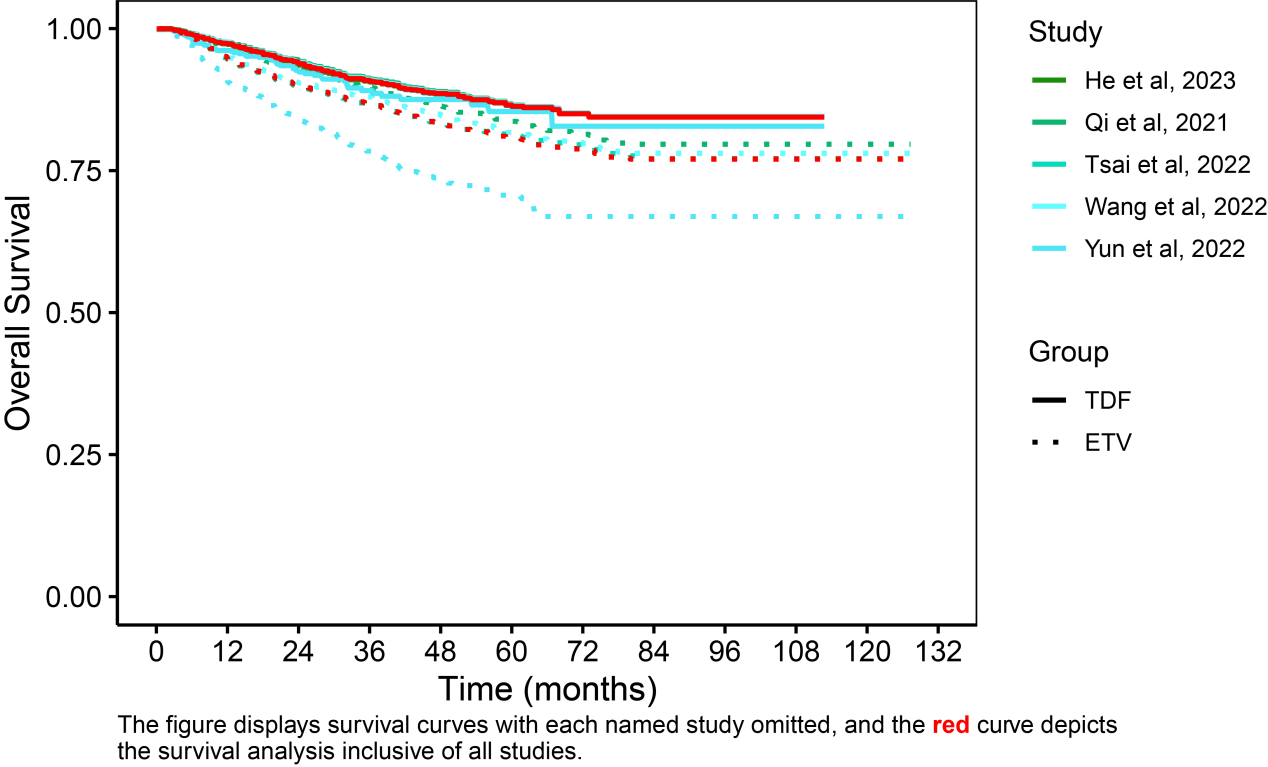
**

**Supplementary Figure S4.** Early and late recurrence in HBV-related HCC patients receiving TDF vs. ETV.

A. Early and late recurrence in HBV-related HCC patients receiving TDF vs. ETV in overall cohort.


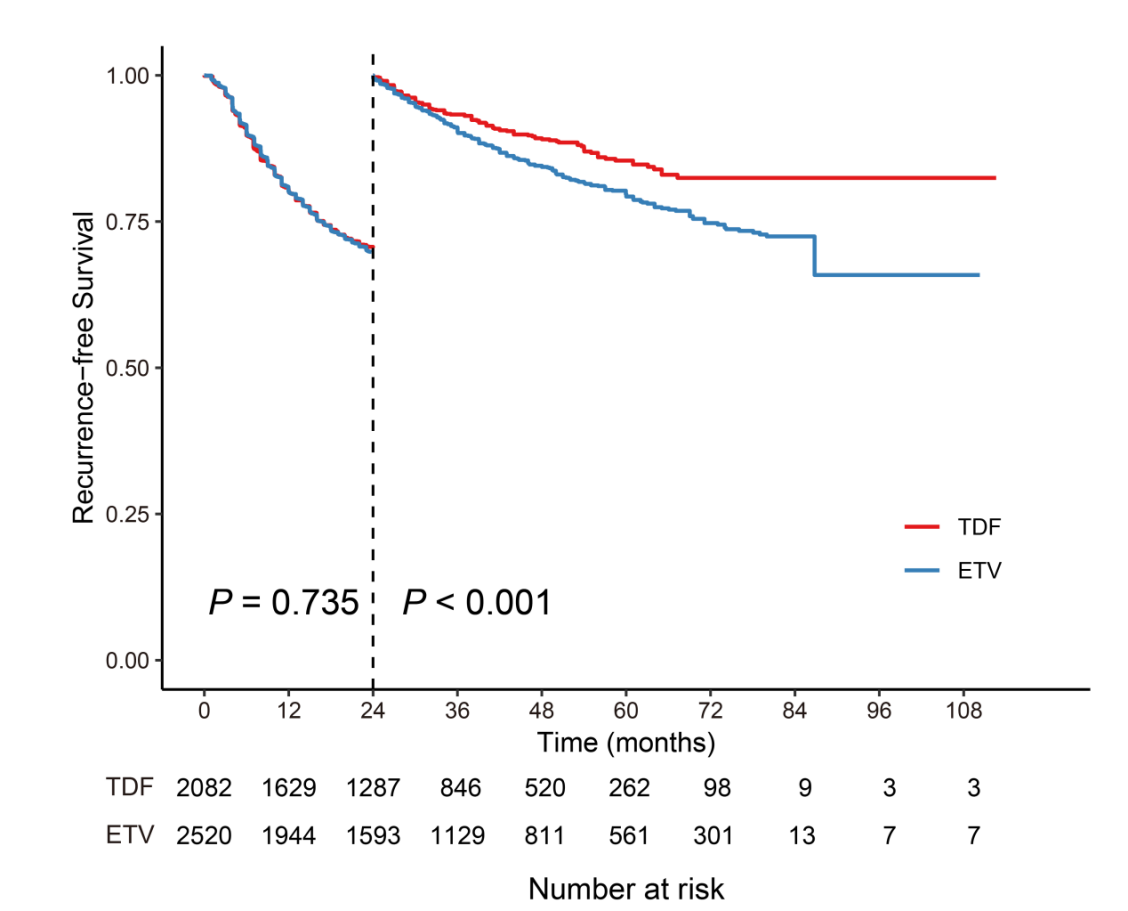


B. Early and late recurrence in HBV-related HCC patients receiving TDF vs. ETV in resection subgroup


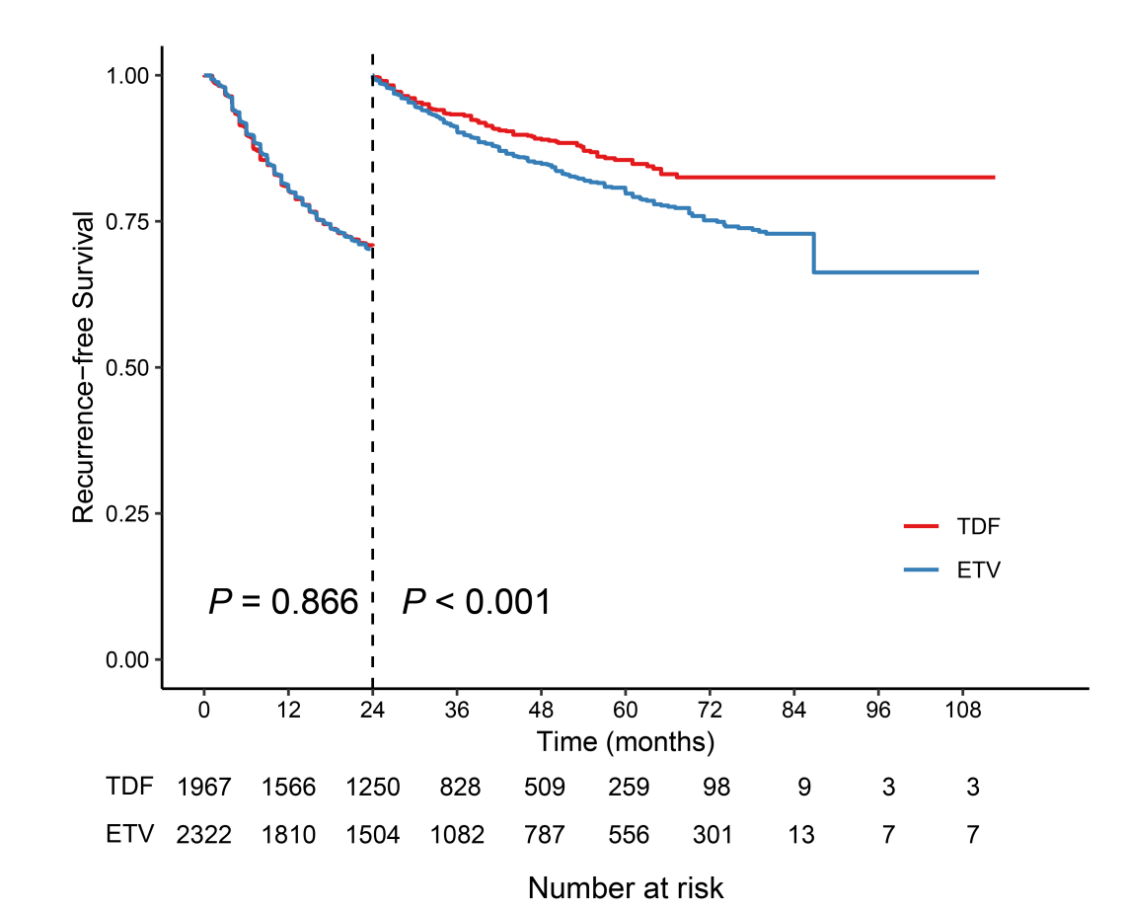


**Figure 1.** Flow Diagram


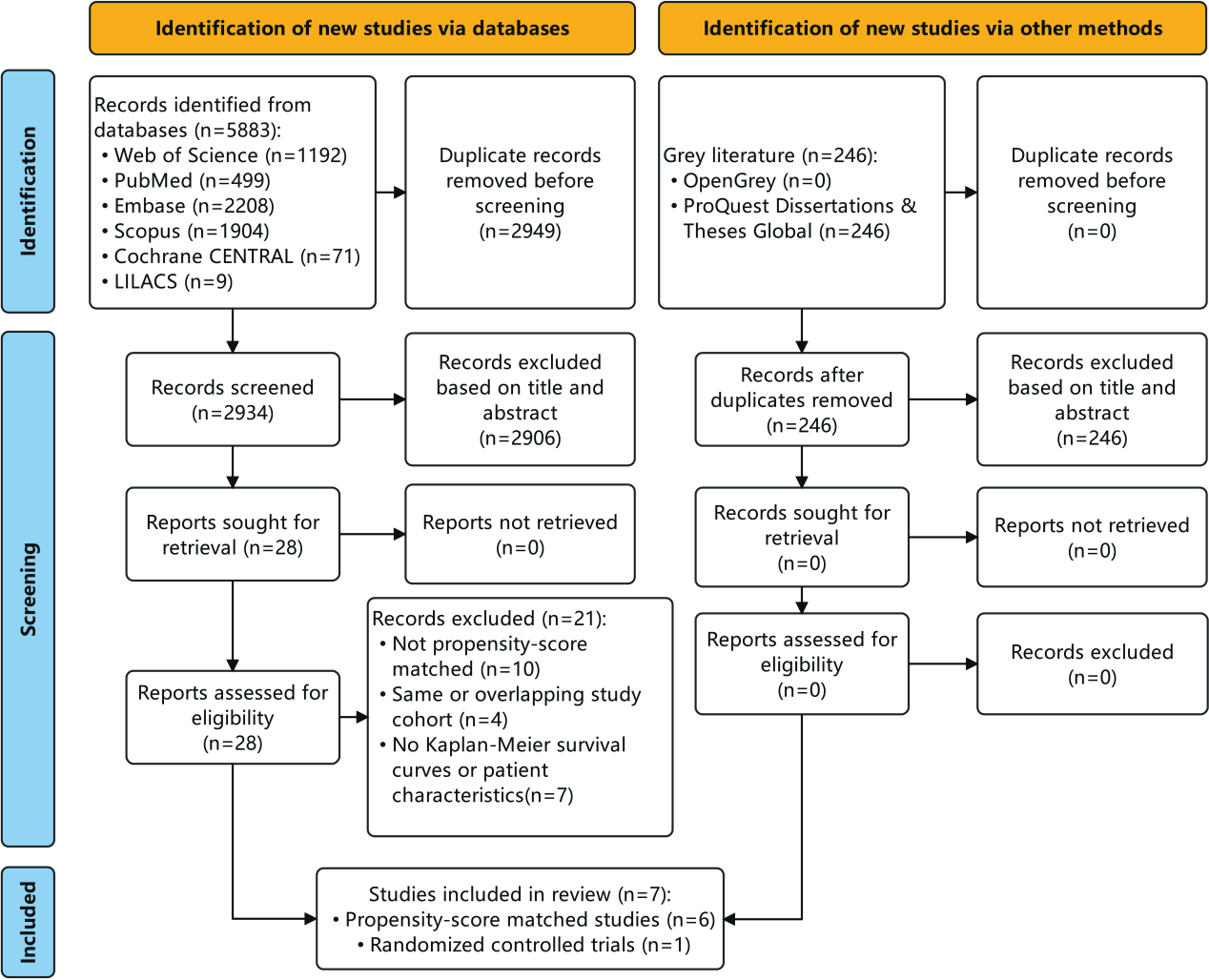


**Figure 2.** Comparison of TDF and ETV in HBV-related HCC patients.


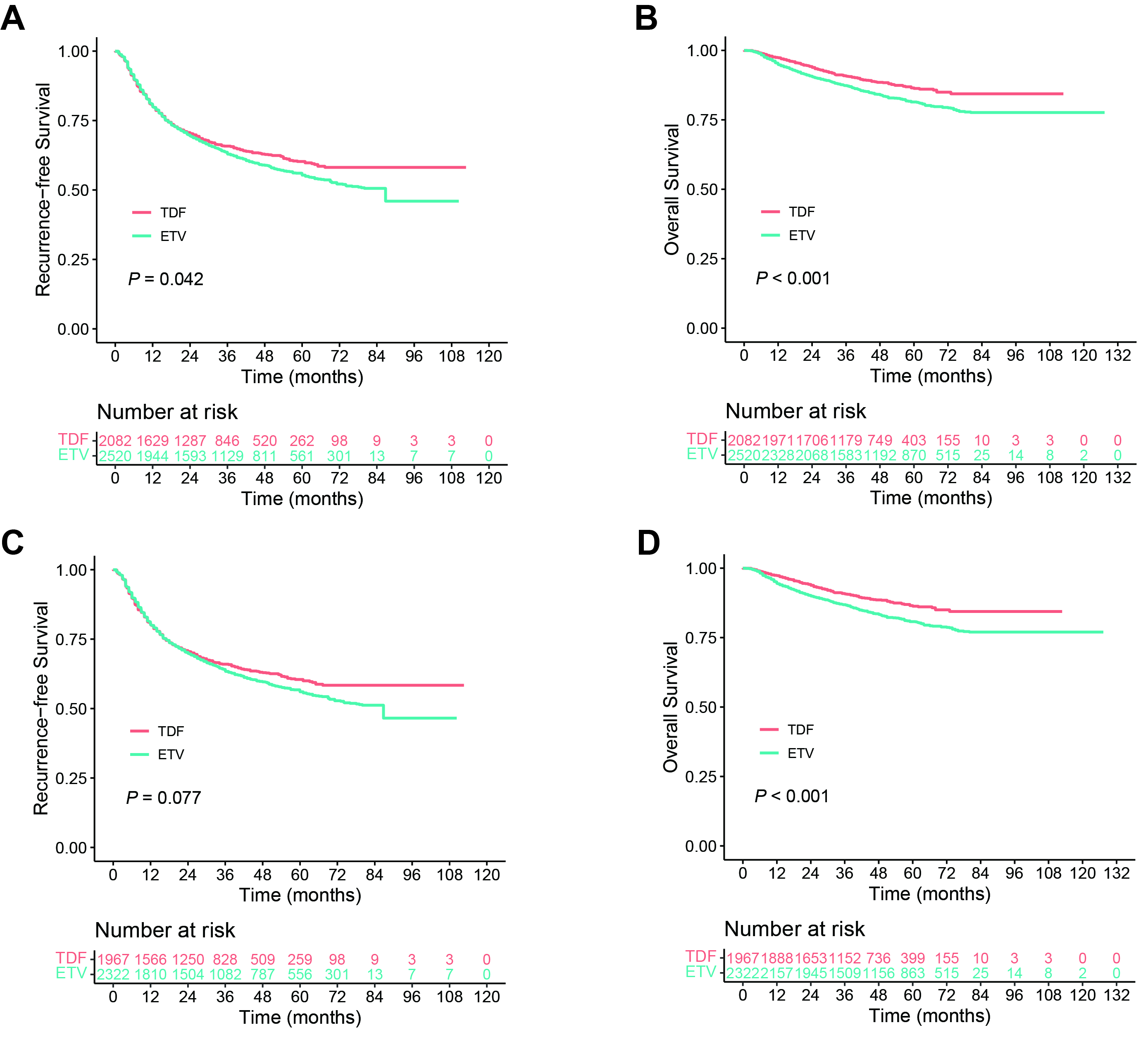


**Figure 2A.** RFS in the overall cohort


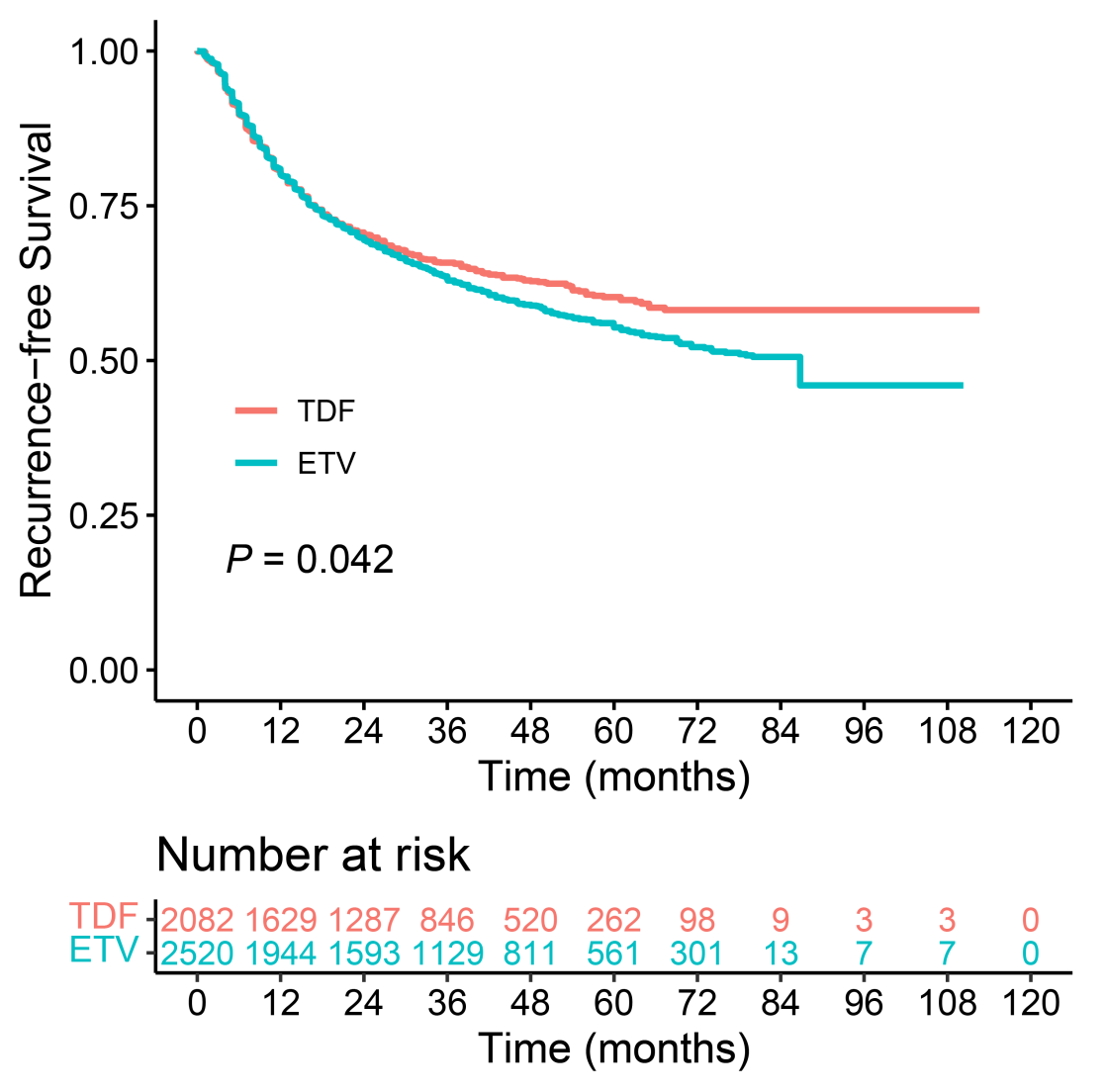


**Figure 2B.** OS in the overall cohort


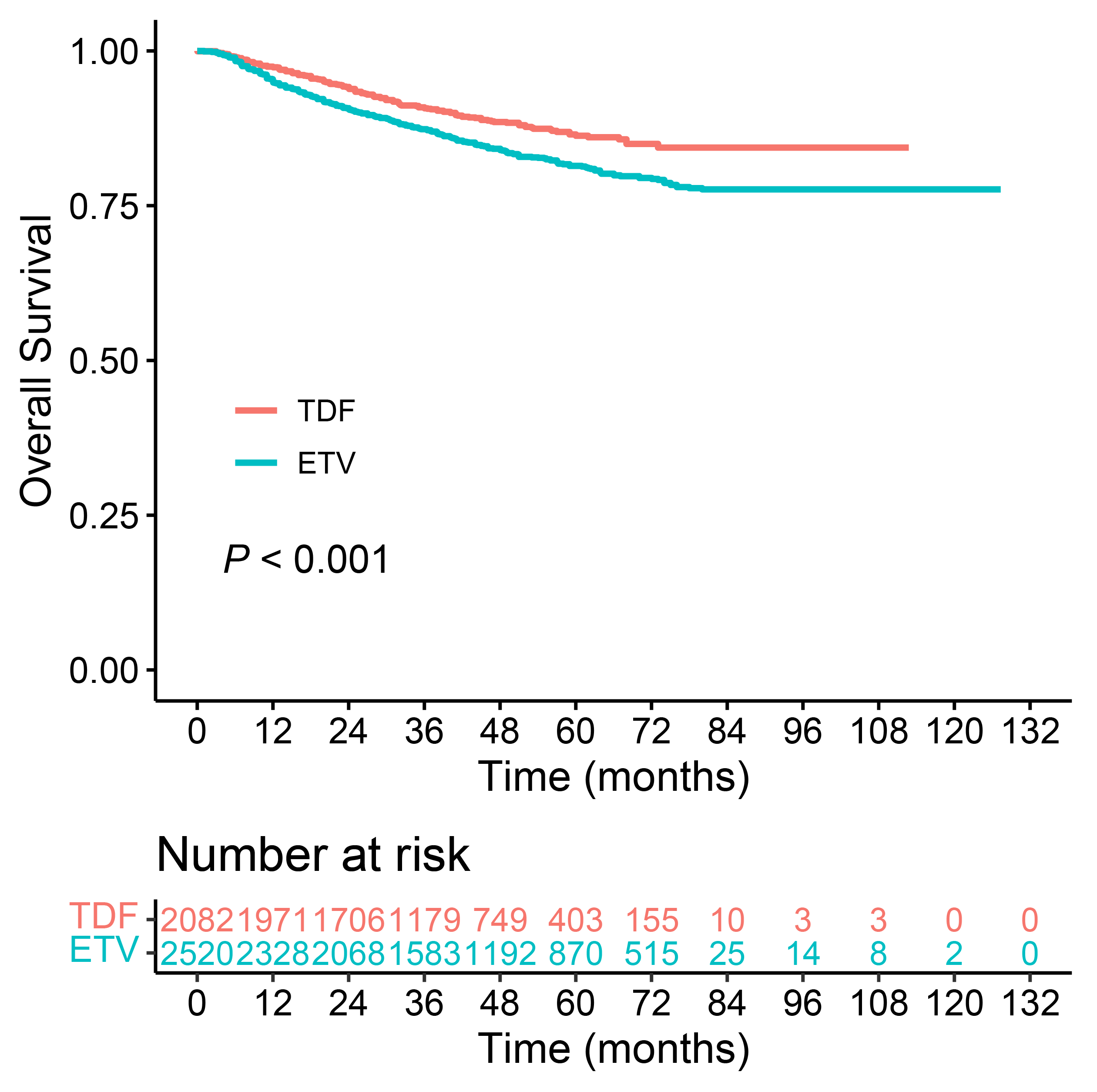


**Figure 2C.** RFS in the Resection Subgroup


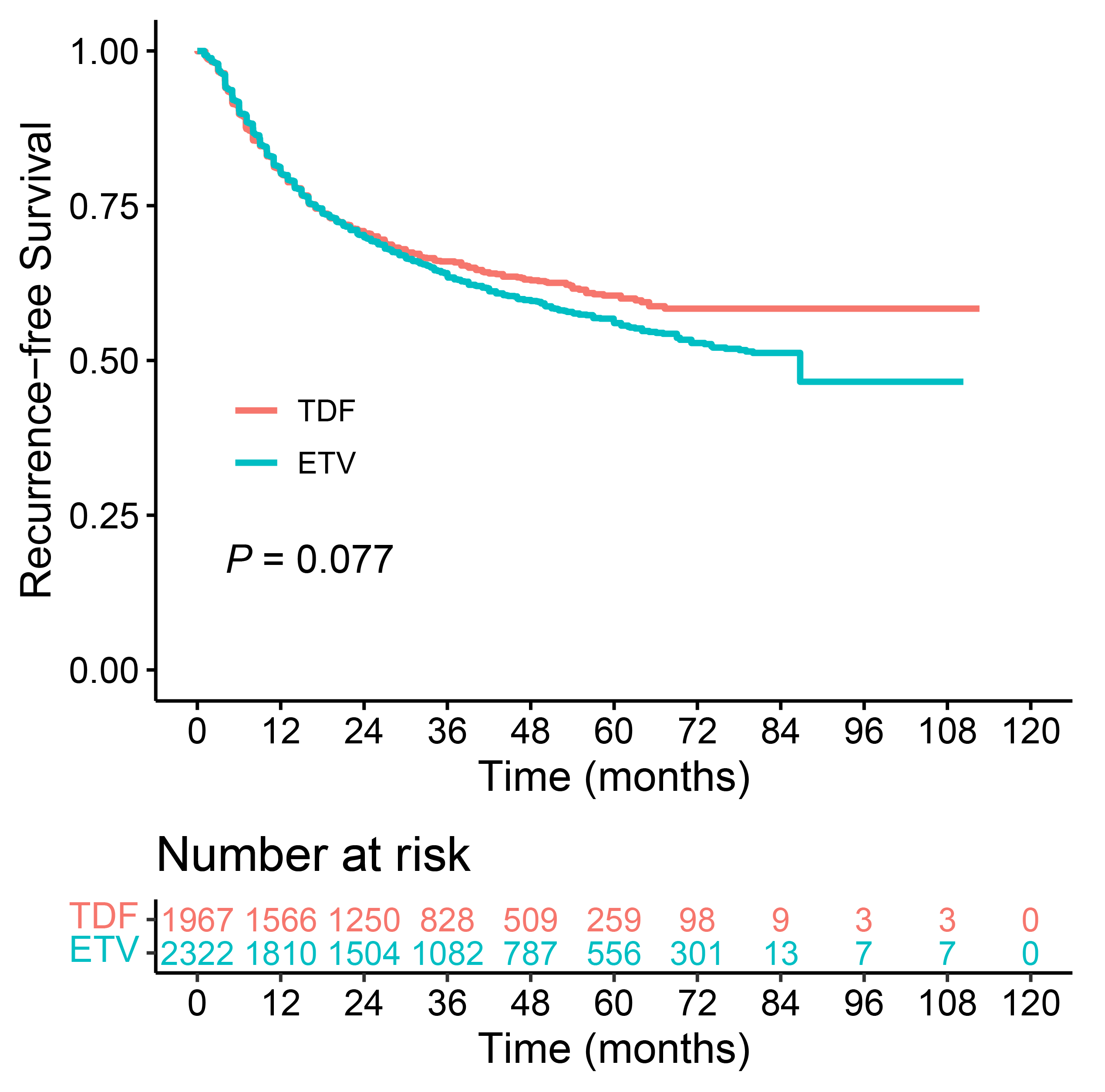


**Figure 2D.** OS in the Resection Subgroup

**
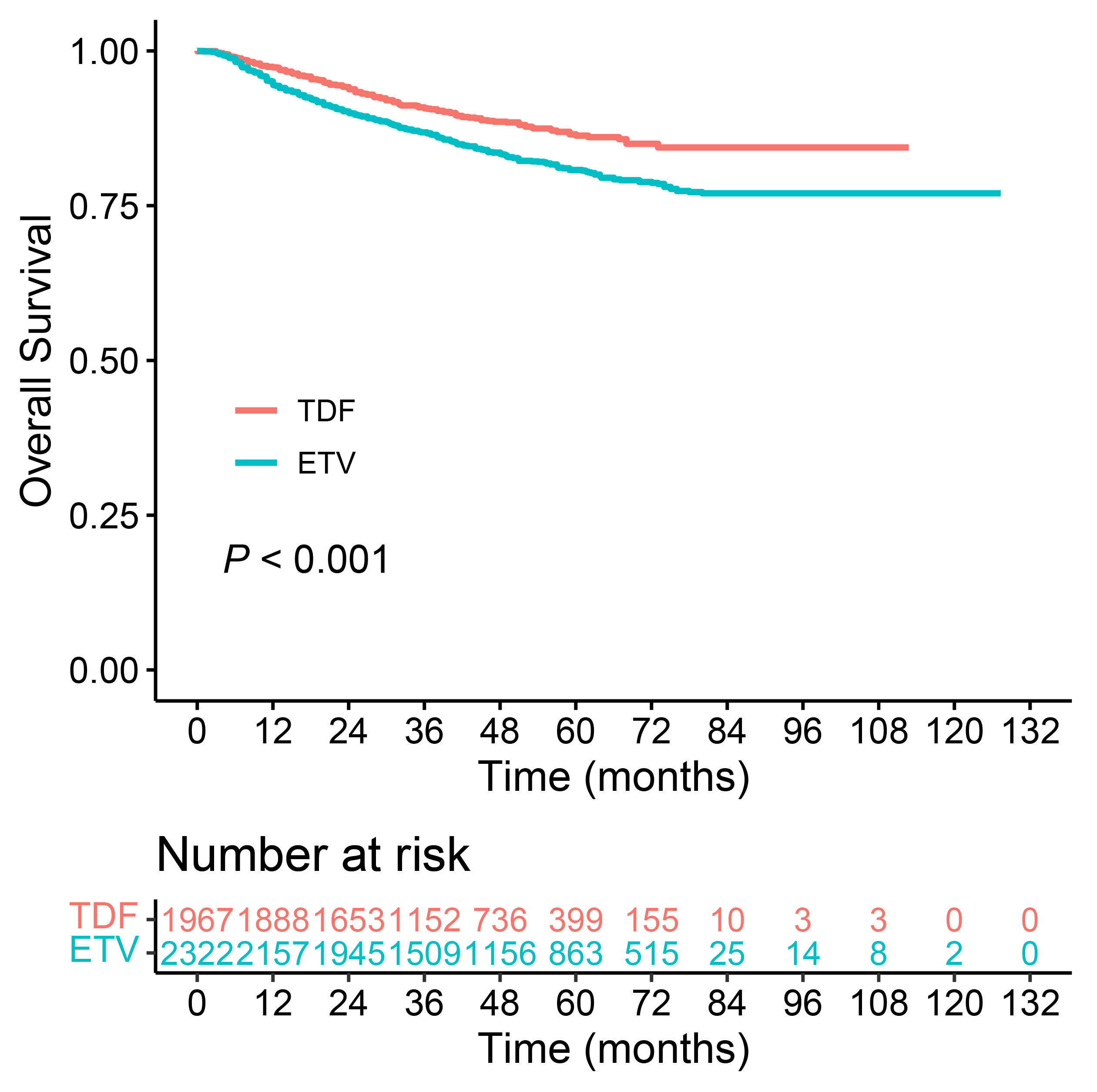
**

**Table 1. Summary of baseline characteristics comparing patients receiving TDF vs. ETV.**

| Characteristics | Number of studies | TDF cohort | |  | ETV cohort | | *P*-value |
| --- | --- | --- | --- | --- | --- | --- | --- |
|  |  | Total number of patients | Value (95% CI) |  | Total number of patients | Value (95% CI) |  |
| Basic characteristics |  |  |  |  |  |  |  |
| Age, year, mean | 7 | 2082 | 52.38 (50.15-54.60) |  | 2520 | 52.44 (50.11-54.76) | 0.385 |
| Sex, % |  |  |  |  |  |  | 0.087 |
| Male | 7 | 2082 | 86.36 (82.72-90.00) |  | 2520 | 85.68 (81.94; 89.42) |  |
| Female | 7 | 2082 | 13.64 (10.00-17.28) |  | 2520 | 14.32 (10.58-18.06) |  |
| Hypertension, % | 5 | 1971 | 19.74 (10.25-29.22) |  | 2306 | 21.20 (13.83-28.57) | 0.978 |
| Diabetes, % | 6 | 2044 | 13.89 (8.49-19.28) |  | 2452 | 15.87 (11.57-20.17) | 0.396 |
| Liver cirrhosis, % | 6 | 2044 | 69.59 (59.09-80.08) |  | 2452 | 68.58 (57.29-79.86) | 0.814 |
| Tumor characteristics |  |  |  |  |  |  |  |
| Tumor size, cm, mean | 6 | 671 | 4.21 (2.31-6.12) |  | 1109 | 4.20 (2.35-6.04) | 0.917 |
| Singe tumor, % | 5 | 633 | 87.45 (79.46-95.45) |  | 1041 | 87.63 (84.29-90.98) | 0.848 |
| Microvascular invasion, % | 5 | 594 | 28.37 (21.92-34.82) |  | 979 | 31.39 (22.70-40.08) | 0.483 |
| Satellite nodule, % | 4 | 329 | 6.37 (3.75-8.99) |  | 576 | 7.80 (3.46-12.15) | 0.930 |
| BCLC stage 0, % | 5 | 633 | 16.23 (1.21-31.24) |  | 1041 | 12.31 (2.02-22.61) | 0.114 |
| High differentiation | 5 | 594 | 10.44 (3.45-31.62) |  | 979 | 7.81 (1.80-33.97) | 0.998 |
| Virologic characteristics |  |  |  |  |  |  |  |
| HBV-DNA ≥ 2000 IU/mL, % | 3 | 412 | 40.33 (25.56-55.09) |  | 623 | 40.24 (23.29-57.18) | 0.814 |
| HBeAg positive, % | 4 | 556 | 23.78 (18.87-28.70) |  | 911 | 23.73 (17.85-29.62) | 0.937 |
| Laboratory Test |  |  |  |  |  |  |  |
| AFP > 20 ng/mL, % | 4 | 489 | 52.30 (47.28-57.33) |  | 753 | 51.20 (45.21-57.20) | 0.885 |
| PLT, 10^9^/L, mean | 4 | 560 | 157.02 (102.11-211.93) |  | 895 | 155.70S(96.62- 214.79) | 0.683 |
| ALT, U/L, mean | 5 | 633 | 43.80 (34.54-53.06) |  | 1041 | 43.29 (34.12-52.46) | 0.725 |
| AST, U/L, mean | 5 | 633 | 41.86 (31.86-51.86) |  | 1041 | 41.27 (31.26-51.28) | 0.548 |
| ALB, g/L, mean | 6 | 671 | 41.21 (39.03-43.40) |  | 1109 | 40.90 (38.80-42.99) | 0.271 |
| TBIL, μmol/L, mean | 6 | 671 | 14.52 (13.92-15.12) |  | 1109 | 16.37 (13.19-19.55) | 0.766 |
| Cr, μmol/L, mean | 3 | 376 | 75.91 (70.29-81.52) |  | 617 | 75.01 (72.08-77.95) | 0.800 |
| PT, s, mean | 4 | 524 | 13.22 (11.37-15.07) |  | 889 | 13.20 (11.57-14.83) | 0.896 |

TDF, tenofovir; ETV, entecavir; CI, confidence interval; BCLC, Barcelona clinic liver cancer; HBV, hepatitis B virus; HBeAg, hepatitis B e antigen; AFP, alpha-fetoprotein; PLT, platelet count; ALT, alanine aminotransferase; AST, aspartate aminotransferase; ALB, albumin; TBIL, total bilirubin; Cr, creatinine; PT, prothrombin time.

**Table 2. Summary of the analysis on early and late recurrence and OS in patients treated with TDF and ETV.**

| Variable | Early recurrence | |  | Late recurrence | |  | OS | |
| --- | --- | --- | --- | --- | --- | --- | --- | --- |
|  | HR (95% CI) | *P*-value |  | HR (95% CI) | *P*-value |  | HR (95% CI) | *P*-value |
| Overall cohort |  |  |  |  |  |  |  |  |
| Stratiﬁed Cox regression | 1.045 (0.937-1.165) | 0.434 |  | 0.681 (0.550-0.843) | <0.001 |  | 0.756 (0.639-0.896) | 0.001 |
| Shared frailty model | 1.040 (0.933-1.160) | 0.480 |  | 0.690 (0.558-0.853) | <0.001 |  | 0.753 (0.636-0.891) | 0.001 |
| Resection cohort |  |  |  |  |  |  |  |  |
| Stratiﬁed Cox regression | 1.050 (0.938-1.176) | 0.393 |  | 0.691 (0.556-0.859) | 0.001 |  | 0.736 (0.620-0.874) | <0.001 |
| Shared frailty model | 1.047 (0.935-1.172) | 0.420 |  | 0.701 (0.565-0.870) | 0.001 |  | 0.731 (0.616-0.868) | <0.001 |

overall survival OS; TDF, tenofovir; ETV, entecavir; CI, confidence interval.
